# Modulation of Oxidative Stress and Apoptosis by *Antrodia cinnamomea*–Loaded Citrate-Stabilized Silver Nanoparticles in Experimental Parkinsonism

**DOI:** 10.1007/s12035-026-05853-5

**Published:** 2026-04-21

**Authors:** Deniz Tekiner, Semin Gedikli, Volkan Gelen, Cemil Bayram, Adem Kara

**Affiliations:** 1 Department of Histology and Embryology, Faculty of Veterinary, Atatürk University, Erzurum, Türkiye; 2 Department of Physiology, Faculty of Veterinary, Kafkas University, Kars, Türkiye; 3 Department of Pharmacology and Toxicology, Faculty of Veterinary, Atatürk University, Erzurum, Türkiye; 4 Department of Molecular Biology and Genetics, Faculty of Science, Erzurum Technical University, Erzurum, Türkiye

**Keywords:** 6-OHDA, *Antrodia cinnamomea*, Oxidative stress, Apoptosis, Neuroprotection, Silver nanoparticles

## Abstract

Parkinson’s disease (PD) is a progressive neurodegenerative disorder characterized by dopaminergic neuron loss in the substantia nigra pars compacta, accompanied by oxidative stress and neuroinflammation. Novel multitarget neuroprotective strategies are required to overcome the limitations of current symptomatic treatments. The neuroprotective effects of *Antrodia cinnamomea* (AC) and citrate-stabilized silver nanoparticles (AgNPs), alone and in combination, were evaluated using a 6-hydroxydopamine (6-OHDA)–induced SH-SY5Y cell model and a unilateral 6-OHDA rat model. Sixty-three rats were divided into nine experimental groups. Cell viability, behavioral tests, LC–MS/MS analysis of dopamine and acetylcholine, oxidative stress and inflammatory biomarkers, histopathological assessment, immunohistochemistry, and Western blot analyses of TH, α-synuclein, PI3K, Bcl-2, Caspase-3, and agmatinase were performed. 6-OHDA significantly reduced cell viability, impaired motor performance, and induced dopaminergic neuronal degeneration. AC treatment, particularly in combination with AgNPs, markedly improved cell survival, ameliorated behavioral deficits, and preserved neuronal architecture. Combined treatment significantly decreased MDA, TNF-α, and IL-1β levels, while restoring GSH and SOD activities. LC–MS/MS analysis demonstrated partial recovery of dopamine and acetylcholine levels. Increased TH and PI3K expression, reduced α-synuclein and Caspase-3 levels, and normalization of Bcl-2 and agmatinase were observed following AC + AgNP treatment. AC conjugated with citrate-stabilized AgNPs exerts significant neuroprotective effects in experimental PD by concurrently modulating oxidative stress, neuroinflammation, and apoptotic pathways, highlighting its potential as a multitarget therapeutic strategy.

**Graphical Abstract:**

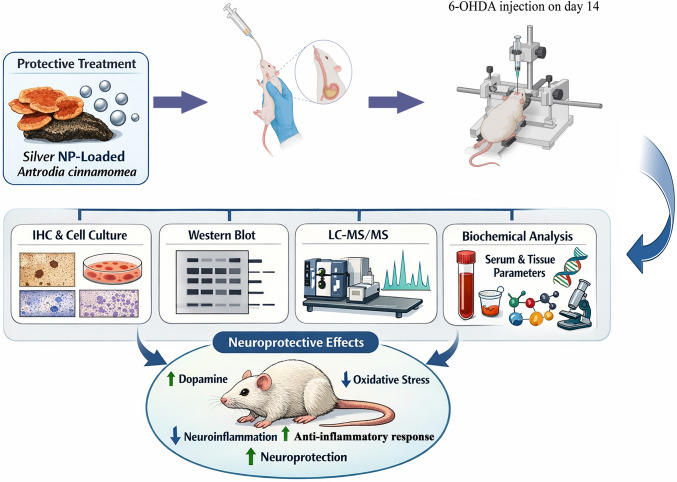

**Supplementary Information:**

The online version contains supplementary material available at 10.1007/s12035-026-05853-5.

## Introduction

Parkinson’s disease (PD) constitutes a major and rapidly expanding global neurological burden, with epidemiological data indicating a continuous rise in incidence and prevalence driven predominantly by population aging and increased life expectancy [1]. The principal pathogenic characteristic of the disease is the gradual degeneration of dopaminergic neurons in the substantia nigra pars compacta, leading to a dopamine deficit in the striatum. This neurochemical imbalance clinically presents as primary motor symptoms, including bradykinesia, stiffness, tremor, and postural instability, with non-motor symptoms such as depression, sleep difficulties, autonomic dysfunction, and cognitive loss [2]. The complex character of Parkinson’s disease etiology is now broadly acknowledged.

Oxidative stress, mitochondrial dysfunction, neuroinflammation, and abnormalities in protein homeostasis are key processes driving the disease [3, 4]. Dopaminergic neurons of the substantia nigra exhibit heightened susceptibility to oxidative damage owing to their elevated oxygen utilization, restricted antioxidant capabilities, and the inherent tendency of dopamine to undergo auto-oxidation [5]. Inhibition of mitochondrial complex I leads to energy failure and promotes the generation of reactive oxygen species (ROS), thereby accelerating lipid peroxidation and compromising neuronal integrity [6].

Protein aggregation, especially the buildup of α-synuclein and the formation of Lewy bodies, constitutes a hallmark pathogenic characteristic of Parkinson’s disease. Impairment of intracellular protein quality-control mechanisms that govern proteolytic degradation and vesicular turnover hinders the effective elimination of misfolded proteins, thereby promoting the accumulation of neurotoxic aggregates [7]. Concurrently, chronic neuroinflammation, along with microglial activation, accelerates neuronal degeneration by increasing proinflammatory cytokine levels, such as TNF-α and IL-1β, thereby intensifying the disease’s progressive traits [8].

Most modern therapy methods rely on replacing dopamine, especially by giving levodopa. This method only helps with symptoms; it does not stop the disease from getting worse. Long-term use can also cause motor fluctuations and dyskinesias [9]. As a result, it is very important to find new neuroprotective agents that work on the main pathophysiological pathways of Parkinson’s disease.

Natural products, especially traditional medicinal mushrooms, have garnered significant interest in neurodegenerative disease research due to their numerous bioactive compounds. *Antrodia cinnamomea*, a medicinal fungus native to Taiwan, possesses a variety of terpenoid-derived compounds, complex polysaccharide structures, and redox-active phenolic substances. It has been shown to exhibit substantial antioxidative characteristics, immunomodulatory effects, and cytoprotective actions against apoptosis [10, 11]. However, comprehensive experimental studies investigating the multifaceted pathology of Parkinson’s disease are still limited.

Recent advancements in nanotechnology have paved the way for pioneering methodologies to augment the bioavailability of natural bioactive compounds while simultaneously facilitating targeted delivery to specific sites. Citrate-stabilized silver nanoparticles (AgNPs) have attracted considerable attention due to their stability, biocompatibility, and capacity to mitigate oxidative stress induced by free radicals. Research has demonstrated that silver nanoparticles can traverse the blood–brain barrier, suggesting their potential as carriers for neuroprotective therapeutics [12]. However, the study inadequately investigated the synergistic effects of *Antrodia cinnamomea* alongside the potential modulatory roles of citrate-stabilized AgNPs in relation to oxidative stress and inflammatory mechanisms.

6-Hydroxydopamine (6-OHDA), a commonly used neurotoxin for inducing dopaminergic neuronal degeneration in experimental models, effectively replicates the pathogenesis of Parkinson's disease by triggering oxidative stress, lipid peroxidation, mitochondrial impairment, and inflammatory reactions [13]. Consequently, the 6-OHDA model provides a reliable experimental platform for evaluating novel neuroprotective approaches. The objective of this study was to examine the effects of *Antrodia cinnamomea*, citrate-coated silver nanoparticles, and their combination on oxidative stress, inflammation, and apoptosis in a 6-OHDA-induced experimental model of Parkinson's disease. The primary hypothesis of this study posits that the concomitant administration of these two agents would engender a more potent and comprehensive neuroprotective response than their separate use.

## Materials and Methods

### Chemicals

The following chemicals were supplied and used for the analyses conducted in this study: 6-hydroxydopamine (Sigma Co., H4381, USA), retinoic acid (Sigma Co., R2625, USA), apomorphine HCl (EPAMOR, Koro İlac, Ankara, Türkiye), silver nitrate (Sigma Co., 209,139, USA), serum IL-1β (YL Bion, Cat. No: YLA0030RA, Shanghai, China), TNF-α (YL Bion, Cat. No: YLA0118RA, Shanghai, China), *Antrodia Cinnamomea*-AC (Taiwan Leader Biotech Corp., 40201005, Taiwan), GSH (YL Bion, Cat. No: YLA0121RA, Shanghai, China), SOD (YL Bion, Cat. No: YLA0115RA, Shanghai, China), MDA (YL Bion, Cat. No: YLA0029RA, Shanghai, China), dopamine hydrochloride (Macklin D806618, Shanghai, China), and acetylcholine chloride (BLD Pharm BD112299, Shanghai, China).

### In Vitro Studies

#### Synthesis and Physicochemical Characterization of Citrate-Stabilized Silver Nanoparticles

The synthesis of silver nanoparticles (AgNPs) utilized a citrate-mediated reduction method that was adapted from well-established protocols [14]. The successful synthesis of nanoparticles was confirmed by ultraviolet-visible spectrophotometry, which showed a prominent surface plasmon resonance peak at around 427 nm (Supplementary Fig. 1B).

#### Spectroscopic and Physicochemical Analysis of Citrate-Coated Silver Nanoparticles

Infrared spectroscopy was used to identify the surface-bound chemical functionalities of the synthesized nanoparticles. Spectral data were collected within the wavenumber range of 400–4000 cm⁻^1^ using an FT-IR spectrometer (Supplementary Fig. 1A). In addition to infrared analysis, an array of physicochemical characterization methods was employed to obtain a more thorough understanding of the properties of the AgNPs. The optical characteristics of the samples were evaluated through UV–Vis spectroscopy, employing a PerkinElmer Lambda 35 spectrophotometer within a wavelength range of 200–800 nm (Supplementary Fig. 1B). Transmission Electron Microscopy (TEM) analysis was conducted using a Hitachi HighTech-7700 instrument to acquire high-resolution images of the nanoparticles (Supplementary Fig. 1C).

#### Development of Citrate-Functionalized Silver Nanoparticles Loaded with *Antrodia Cinnamomea*

*Antrodia cinnamomea* (AC) was used in the form of Leader *Antrodia cinnamomea* capsules. Each capsule (460 mg) was dissolved in sterile water (SF) to prepare a stock solution. The solution was administered orally to animals at a dose of 100 mg/kg body weight. Separately prepared citrate-stabilized silver nanoparticle (AgNP) suspensions at a concentration of 1 mg/kg were combined with the AC solution at a 1:1 (v/v) ratio and gently agitated under continuous magnetic stirring for approximately 3 h to facilitate surface adsorption driven by electrostatic interactions, enabling loading of the mushroom extract onto the nanoparticles. The resulting formulations were freshly prepared prior to each in vitro and in vivo experimental application [15].

#### In Vitro Parkinson’s Disease Models and Cell Viability Assessment

Differentiated SH-SY5Y cells were plated in 96-well culture plates and allowed to stabilize for 24 h prior to experimental exposure. Treatment compounds and dosage ranges are detailed in the Supplementary Methods section. Cellular metabolic activity, used as an indicator of viability, was quantified using the CCK-8 colorimetric assay (CVDK-8, Ecotech Biotech, Türkiye) in strict accordance with the supplier’s protocol. Optical density values were recorded at 450 nm with a µQuant microplate reader (Biotek). Optimal working concentrations of AC and AgNPs were selected based on viability outcomes obtained from the CCK-8 assay in combination with evidence from earlier investigations [14, 16]. The procedures for establishing the cellular model are described in detail in the Supplementary Methods.

### In Vivo Studies

#### Animal Model and Experimental Procedures

All experimental procedures involving animals were conducted in compliance with institutional ethical guidelines and were approved by the Atatürk University Local Committee for Animal Research (Approval No: 2024/04). Sixty-three adult male Sprague–Dawley rats (12 weeks of age) were supplied by the Atatürk University Experimental Research Center and randomly assigned to nine experimental groups (*n* = 7 per group).

Male Sprague–Dawley rats were selected for their widespread use in neurodegenerative disease models, particularly Parkinson’s disease, and for their well-characterized physiology, which enables reliable behavioral and biochemical analyses [17, 18]. Male animals were preferred to minimize hormonal variability, since estrogen has been reported to exert neuroprotective effects and the estrous cycle in females may influence oxidative stress, apoptosis, and dopaminergic neurodegeneration, potentially affecting experimental outcomes [19, 20]. Animals were maintained in standard laboratory cages, housing four rats per cage, under regulated environmental conditions, including a temperature of 23 ± 2 °C and a 12 h light/dark photoperiod. Standard chow and drinking water were available without restriction throughout the study.

#### Experimental Design

To facilitate in vivo investigations, animals were categorized into nine experimental groups, each comprising seven rodents, and the administration of *Antrodia cinnamomea* was initiated. Body mass was recorded daily throughout the treatment. Citrate-stabilized silver nanoparticles (1 mg/kg) [21] and/or *Antrodia cinnamomea* (AC; 100 mg/kg), obtained as a commercial product (Leader *Antrodia cinnamomea* Capsule; product code: 40201005) from Taiwan Leader Biotech Corp., New Taipei City, Taiwan, consisting of *Antrodia cinnamomea* (*Taiwanofungus camphoratus*) mycelium extract produced by solid-state cultivation technology, were administered for a continuous period of 14 days [22].

On the final day of treatment, Parkinsonian pathology was established by unilateral stereotaxic delivery of 6-hydroxydopamine into the substantia nigra. For detailed stereotaxic 6-OHDA dosing and administration procedures, see Supplementary Experimental Procedures. Rats displaying characteristic unilateral rotational responses were designated as successfully lesioned. Following behavioral confirmation, animals were deeply anesthetized with isoflurane and sacrificed, after which blood specimens were obtained by cardiac puncture. Subsequently, brain tissues were swiftly excised and prepared for subsequent biochemical, histological, and molecular examinations. A detailed account of group-specific interventions is included in the Supplementary Methods.

### Behavioral Tests

#### Forelimb Use Asymmetry Assessment

The subjects’ asymmetrical motor function and spontaneous forelimb activity were evaluated using a standardized vertical exploration paradigm. Each rat was introduced individually into a transparent cylindrical enclosure (200 × 400 mm), and spontaneous behavior was video-monitored for a 30-min observation period. Contacts made by each forelimb against the cylinder surface were systematically counted and expressed as a proportion of the total number of wall contacts. Behavioral scoring was conducted by investigators who were unaware of group allocations. A comprehensive description of the testing protocol is presented in the Supplementary Methods [14, 21].

#### Apomorphine-Evoked Rotational Behavior Analysis

The apomorphine-induced rotational behavior test was employed to verify the successful induction of the Parkinsonian model and to evaluate the effects of the administered treatments [23]. This behavioral assay was performed exclusively in the 6-OHDA-lesioned groups to confirm unilateral dopaminergic denervation; therefore, non-lesioned control groups were not subjected to this test. Prior to behavioral testing, the animals were acclimatized to the experimental apparatus. Apomorphine (2.5 mg/kg) was administered intraperitoneally, and the total number of complete contralateral rotations was recorded over a 30-min observation period. Behavioral assessments were performed by investigators blinded to the group allocation [14, 24]. A detailed description of the protocol is provided in the Supplementary Methods.

#### Locomotor Activity Tests

To perform an objective evaluation of motor function impairments in the Parkinson’s disease model, a locomotor activity test was conducted. The objective of the present study was twofold: first, to use the test to assess the impact of dopaminergic system degeneration on movement patterns and, second, to determine the motor-level efficacy of the applied treatments. Each animal was individually placed into an open-field observation chamber (420 × 420 × 420 mm), and spontaneous activity was recorded for 20 min using the May Act 508 system. A range of parameters was measured automatically, including ambulatory activity, horizontal and vertical movements, stereotyped behaviors, resting time, and total distance traveled. The collected data were analyzed to evaluate the severity of PD-associated motor dysfunctions and the effects of the treatments on motor behavior [25]. A comprehensive description of the experimental procedures is available in the Supplementary Methods section.

### Biochemical Analyses

#### Oxidative Stress–Related Biomarkers

The present study is aimed at investigating the effects of silver nanoparticles incorporating *Antrodia cinnamomea* on oxidative stress–related biomarkers in brain tissue within a 6-hydroxydopamine (6-OHDA)–induced Parkinsonian model. For biochemical analyses, the midbrain region, including the substantia nigra, was carefully dissected from all experimental and control animals. To remove residual blood and debris, tissue specimens were washed with 0.9% physiological saline. Tissue samples (~0.1 g per animal; animals’ average body weight 250 ± 15 g) were then disrupted using a Qiagen TissueLyser II in the presence of liquid nitrogen at 30 Hz for 3 min, followed by homogenization in an appropriate buffer for 30 s using the same device.

Oxidative stress markers—malondialdehyde (MDA), reduced glutathione (GSH), and superoxide dismutase (SOD)—were quantified and normalized to total protein content determined by the Bradford method [26]. Lipid peroxidation, assessed via MDA levels, was measured at 532 nm using the thiobarbituric acid (TBA) method as previously described [27]. GSH levels were determined spectrophotometrically by measuring the formation of 5-thio-2-nitrobenzoic acid (TNB) from DTNB at 412 nm [28]. SOD activity was analyzed using a method that measures inhibition of nitroblue tetrazolium (NBT) reduction [29].

This approach ensured sufficient tissue quantity for accurate biochemical measurements, maintained methodological consistency across all groups, and minimized variability arising from microdissection of small anatomical regions, such as the substantia nigra.

#### LC–MS/MS Analysis

Dopamine and acetylcholine levels in tissue samples obtained from the substantia nigra of rats were analyzed to quantify alterations in dopaminergic and cholinergic neurotransmission in the experimental Parkinsonian model [30, 31]. These neurotransmitters are closely associated with motor and cognitive functions, making their levels crucial indicators for understanding disease pathogenesis and evaluating treatment effectiveness [32, 33]. Substantia nigra tissue samples were meticulously handled under controlled conditions and immediately frozen in liquid nitrogen to prevent oxidative degradation. Samples were mechanically disrupted under cryogenic conditions, and acetonitrile (Isolab, 901.037.2501, Germany) was used as both the homogenization and extraction solvent. Homogenization was performed using stainless steel beads in a TissueLyser II system (Qiagen, Germany). Following homogenization, samples were centrifuged, and the resulting supernatants were collected for analysis. Neurotransmitter concentrations were quantified using a highly sensitive and selective liquid chromatography-tandem mass spectrometry (LC–MS/MS) methodology. Calibration curves were developed to enhance analytical precision, and these were applied in the subsequent quantitative analysis. This procedure ensured efficient protein precipitation, optimal recovery of neurotransmitters, and reproducibility across all experimental groups.

#### Histopathological Analysis

A complete examination of neurodegeneration was conducted using hematoxylin and eosin staining on 5 μm sagittal slices of the substantia nigra (SN). Identification of the SN was confirmed based on Paxinos and Watson atlas coordinates [34] and characteristic anatomical landmarks. Two independent investigators, blinded to the experimental groups, assessed neuronal damage by calculating the ratio of damaged neurons to the total neuronal cell count. The semiquantitative scoring method used was described previously [35].

#### Immunohistochemical Analysis

Immunostaining procedures were modified based on the protocols reported by Özmen and Haligur [36]. Antigen unmasking was performed using a citrate-based retrieval solution adjusted to pH 6.0 using four heating cycles at 600 W, each lasting 5 min. After completion of this procedure, tissue sections were treated with the blocking solution provided in the HRP detection kit (PatoLab HRP PL-125-HL, PatoLab Biotechnology, Türkiye) to minimize nonspecific binding, then incubated at 4 °C for an extended period with the primary antibodies specified in the Supplementary Methods. Secondary immunoreactivity was detected using an anti-polyvalent large-volume detection system, and antigen–antibody binding was rendered visible through chromogenic development with 3,3′-diaminobenzidine (DAB; Thermo Fisher Scientific). After completion of staining, sections were examined and imaged under appropriate magnifications using a Leica DM2500 (Germany) binocular light microscope. Immunostaining signals were first verified against negative control sections and were subsequently quantified in the substantia nigra using the immunoreactive scoring (IRS) approach.

#### Semi-Quantitative Analysis of Immunoreactive Signals

This method is a semiquantitative scoring system used to evaluate protein expression in immunohistochemical staining. The total score, ranging from 0 to 12, is obtained by multiplying staining intensity (0–3) by the percentage of positive cells (0–4) [37]. In the present study, the immunoreactivity of tyrosine hydroxylase (TH) and alpha-synuclein (α-syn) proteins was evaluated using the IRS method. Tissue sections obtained from each rat were examined, and five randomly selected fields per section were analyzed. IRS values were calculated based on the percentage of immunopositive cells and staining intensity (Table 1).

**Table 1:** Immunoreactivity scoring (IRS) evaluation criteria

Positive cell percentage (*A*)	Staining intensity (B)	IRS score (*A* × *B*)	Immunoreactivity level
0—no positive cells	0—no staining	0–1	Negative
1—positive cells: <10%	1—weak	2–3	Weakly positive
2—positive cells: 10–50%	2—moderate	4–8	Moderately positive
3—positive cells: 51–80%	3—strong	9–12	Strongly positive
4—positive cells: >%80	-	-	-

#### Western Blot Analysis

Brain specimens collected from experimental animals were preserved at −80 °C until further processing. Prior to analysis, tissues were accurately weighed, cryogenically ground in liquid nitrogen, and lysed in radioimmunoprecipitation assay (RIPA) buffer supplemented with protease and phosphatase inhibitor cocktails (Ecotech Biotech, Türkiye). Mechanical homogenization was performed using a Qiagen TissueLyser II (Qiagen, USA) at 30 Hz for 20 s. This rapid homogenization ensured efficient disruption of cell membranes, reproducible protein extraction, and sufficient protein yield for downstream analyses. Protein content was quantified using the Pierce BCA Protein Assay Kit (Thermo Scientific, USA).

For electrophoretic separation, equal amounts of protein (25 µg per lane) were resolved on 10% SDS–polyacrylamide gels and electro transferred onto polyvinylidene difluoride (PVDF) membranes. Membranes were blocked with 5% BSA in PBS-T for 1 h at room temperature to prevent non-specific binding. Primary antibodies were incubated overnight at 4 °C on an orbital shaker, followed by incubation with horseradish peroxidase-conjugated secondary antibodies for 1 h at room temperature. Chemiluminescent detection of immunoreactive bands was conducted using a Western ECL substrate (Thermo, 3405), and densitometric analysis was performed with Image Lab™ software (Bio-Rad, USA).

#### Statistical Analysis

The distributional characteristics of the data informed the statistical analyses. We employed one-way analysis of variance (ANOVA) to compare groups with normally distributed variables. In the event that substantial differences were identified, we employed Dunnett’s post hoc test. We employed the Kruskal–Wallis test for non-normally distributed variables and utilized Dunn’s post hoc multiple comparison test to identify additional differences. Statistical significance was established with a *p*-value below 0.05, and all analyses were performed using GraphPad Prism version 10.1.2.

## Results

### In Vitro Analyses

#### Cytotoxicity Results

**Fig. 1:**
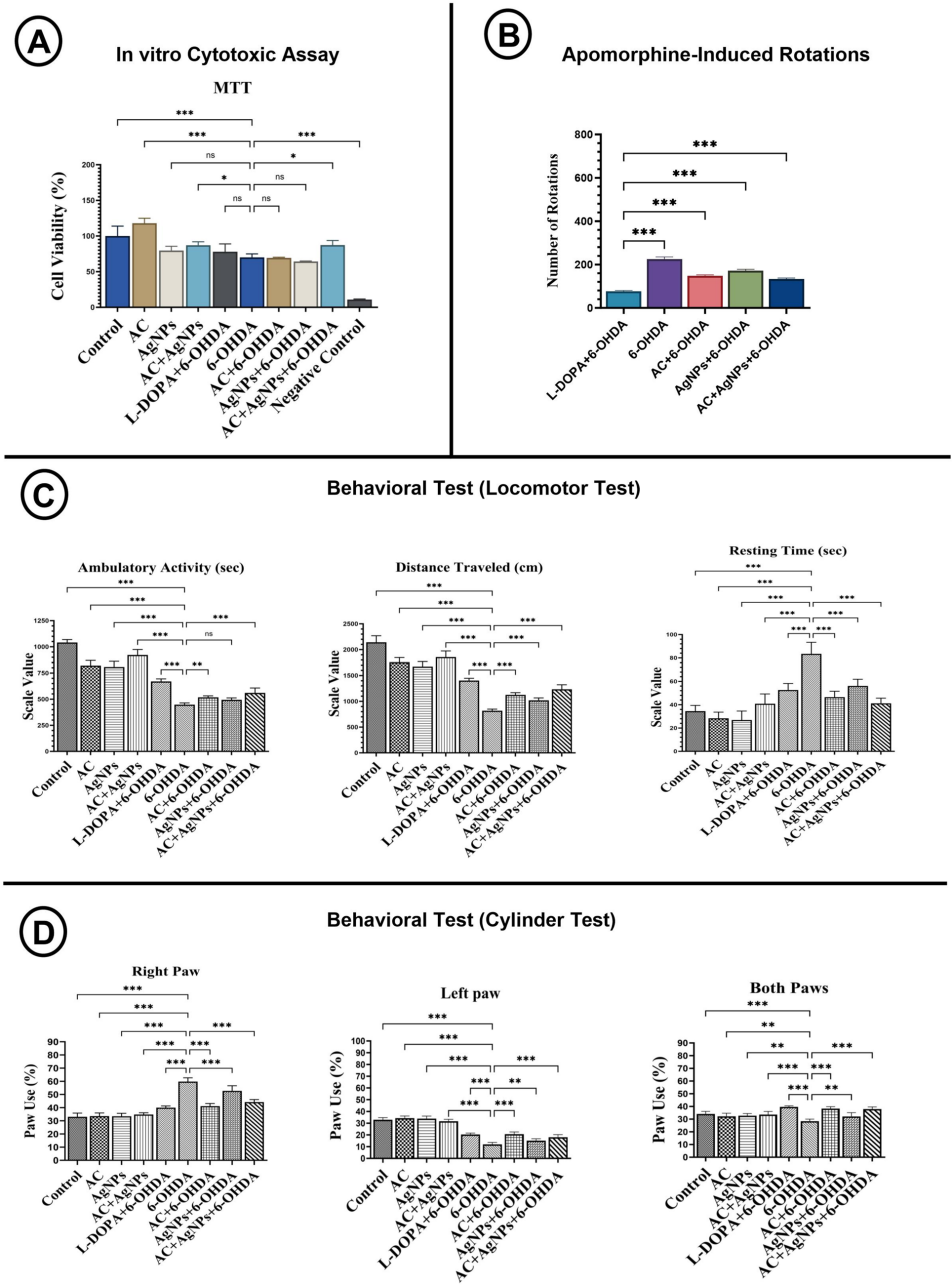
**A** In vitro cytotoxicity evaluation of AC and/or AgNPs on SH-SY5Y neuroblastoma cells in a Parkinson’s disease cellular model. **B** Apomorphine-induced turning behavior (rotameter) test. **C** Locomotor activity was evaluated by assessing ambulatory activity, distance traveled, and resting time per minute over a 30-min test period. **D** Cylinder test percentages of right, left, and both paw touches per 30 min. Statistical significance is indicated by asterisks (*, *p* < 0.05; **, p < 0.01; ***, p < 0.001; ns, p > 0.05)

A noticeable decrease in cell viability was observed following exposure to 6-OHDA. The application of AC and AC + AgNP significantly improved cell viability decreased through 6-OHDA. The triple combination (AC + AgNP + 6-OHDA) enhanced cell viability, indicating a protective effect. However, AgNP treatment alone is associated with reduced cell viability. No significant changes were detected in the other treatment groups (Fig. 1A).

### In Vivo Analyses

#### Behavioral Tests

##### Apomorphine-evoked rotational behavior analysis

This test was performed 48 h after 6-OHDA administration to identify rats exhibiting dopaminergic dysfunction. As the apomorphine-induced rotation test was conducted only in the 6-OHDA-lesioned groups, behavioral assessment was limited to these groups. The number of contralateral rotations was markedly increased in the 6-OHDA group. In contrast, rotation counts were significantly reduced in the AC + 6-OHDA, AgNP + 6-OHDA, and AC + AgNP + 6-OHDA groups compared with the 6-OHDA group. Although rotations were also reduced in the L-DOPA + 6-OHDA group, they remained higher than those observed in the other treatment groups. Apomorphine-induced rotations differed significantly among the lesioned groups (*p* < 0.05). Observers were blinded to group allocation. Rotation counts for the experimental groups are presented in Fig. 1B.

#### Locomotor Activity Test

Locomotor activity was recorded using a computer-assisted analysis system, and group-based activity maps were generated, with horizontal movements indicated by blue lines and vertical movements by green lines. In the control, AC, AgNP, and AC + AgNP groups, movements were widely distributed and regular. In contrast, the 6-OHDA group exhibited a notable reduction in activity, predominantly confined to the periphery. In the L-DOPA + 6-OHDA cohort, activity was augmented and exhibited a distribution akin to that of the control group. In the AC + 6-OHDA and AgNP + 6-OHDA groups, movements were more broadly distributed than in the 6-OHDA group, while the AC + AgNP + 6-OHDA group demonstrated the most vigorous and extensive exploratory activity. A behavioral test for the experimental groups is presented in Fig. 1C, and the locomotor activity maps for the experimental cohorts are illustrated in Supplementary Fig. 1A.

The quantitative analysis of locomotor test results encompassed a variety of parameters including ambulatory movements, total distance traversed, and duration of rest. The 6-OHDA group demonstrated a marked decline in ambulatory movements, the shortest total distance traveled, and the longest duration of rest. In the AC + 6-OHDA and AC + AgNP + 6-OHDA groups, ambulatory movements and total distance increased, accompanied by reduced resting time. No marked changes in movement or distance were observed in the AgNP + 6-OHDA group. In the control, AC, AgNP, and AC + AgNP groups, locomotor activity and total distance were high, while resting time remained low. Group-wise comparisons of ambulatory movements, total distance traveled, and resting time are presented in Fig. 1C.

#### Forelimb Use Asymmetry Assessment

Following 14 days of treatment, Parkinson’s disease was induced by 6-OHDA, and the effects of neurodegeneration on forelimb use were evaluated. In the 6-OHDA group, right forelimb use was significantly increased compared with all other groups, whereas left forelimb and simultaneous (both forelimbs) use were markedly reduced. In the AgNP + 6-OHDA group, left forelimb use increased relative to the 6-OHDA group. The percentage of simultaneous forelimb use was reduced in the 6-OHDA group compared with the control, AC + AgNP, L-DOPA + 6-OHDA, AC + 6-OHDA, and AC + AgNP + 6-OHDA groups, and a notable decrease in simultaneous forelimb use was also observed in the AC, AgNP, and AgNP + 6-OHDA groups. Percentages of simultaneous forelimb use are statistically summarized in Fig. 1D.

### Biochemical Analyses

#### Oxidative Stress–Related Biomarkers

To determine levels of oxidative stress–related parameters in rats with an experimentally induced Parkinson’s disease model, malondialdehyde (MDA), reduced glutathione (GSH), and superoxide dismutase (SOD) levels were analyzed in brain tissues. Group differences in MDA, GSH, and SOD levels were assessed. MDA concentrations were significantly increased in the 6-OHDA-treated group relative to the remaining experimental groups. Relative to the 6-OHDA group, MDA levels were significantly reduced in the AC, AgNP, AC + 6-OHDA, AC + AgNP, L-DOPA + 6-OHDA, AgNP + 6-OHDA, and AC + AgNP + 6-OHDA groups. With respect to GSH levels, the 6-OHDA group exhibited markedly lower values than those in the control, AC, AgNP, and AC + AgNP groups (*p* < 0.001). No significant differences were observed between the 6-OHDA group and the L-DOPA + 6-OHDA, AC + 6-OHDA, or AgNP + 6-OHDA groups (*p* > 0.05). In contrast, GSH levels were significantly increased in the AC + AgNP + 6-OHDA group compared with the 6-OHDA group (*p* < 0.05).

**Fig. 2:**
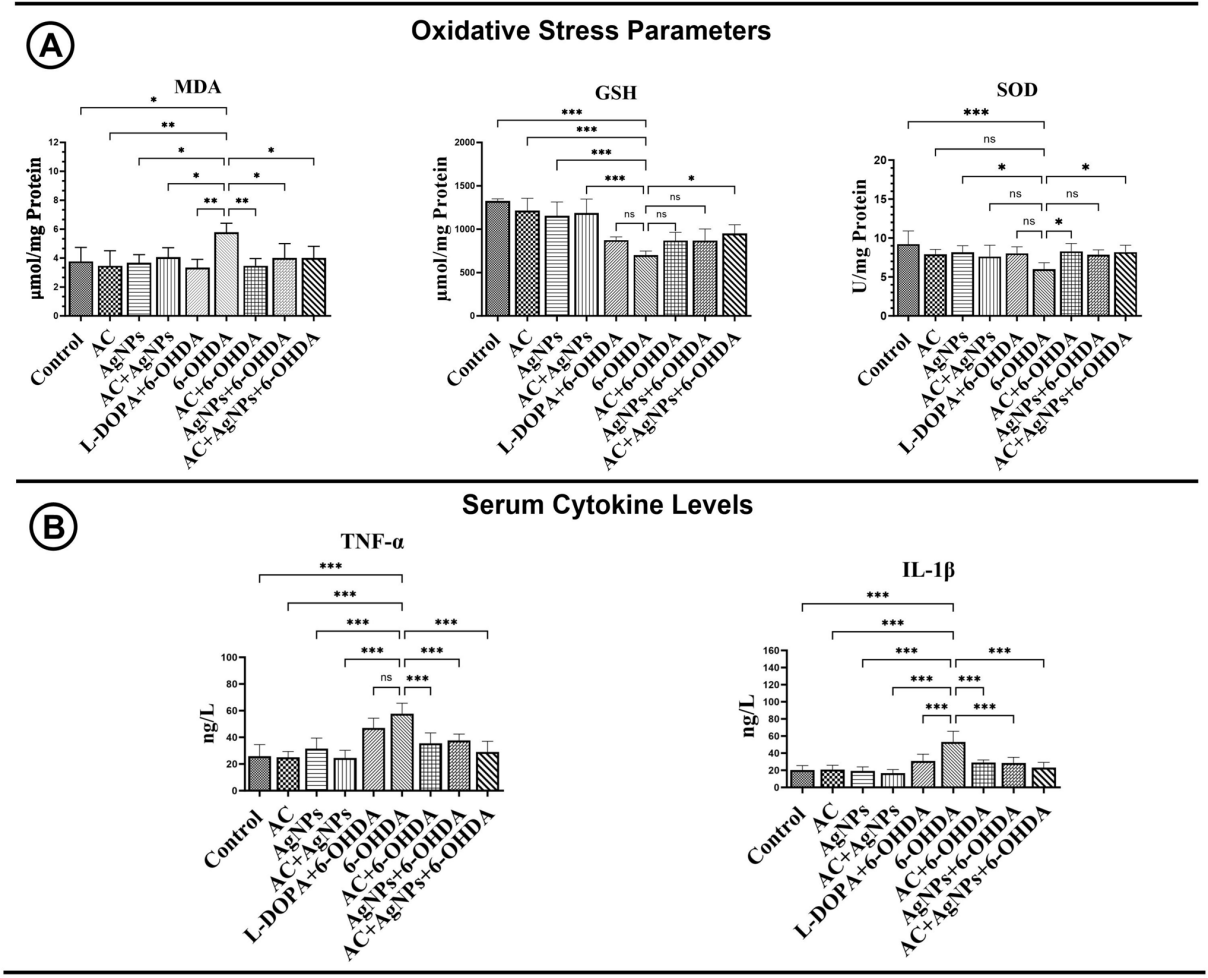
**A** Brain tissue levels of malondialdehyde (MDA), glutathione (GSH), and superoxide dismutase (SOD). **B** Serum levels of IL-1β and TNF-α. All data were analyzed using one-way ANOVA followed by Tukey’s post hoc test. Statistical significance is indicated by asterisks (*p < 0.05, **p < 0.01, ***p < 0.001 and ns: *p* > 0.05)

A study of superoxide dismutase (SOD) levels revealed that the 6-OHDA group exhibited significantly reduced SOD activity compared to both the AgNP group (*p* < 0.05) and the control group (*p* < 0.001). SOD levels were found to be significantly elevated in the AC + 6-OHDA and AC + AgNP + 6-OHDA groups in comparison to the 6-OHDA group (*p* < 0.05). No substantial differences were observed between the 6-OHDA group and the AC, AC + AgNP, L-DOPA + 6-OHDA, or AgNP + 6-OHDA groups (*p* > 0.05). As illustrated in Fig. 2A, the analytical results of serum MDA, SOD, and GSH levels for all experimental groups are presented.

### Serum Cytokine Analyses

The systemic inflammatory response was assessed by quantifying serum levels of tumor necrosis factor-alpha (TNF-α) and interleukin-1 beta (IL-1β). The TNF-α levels in the 6-OHDA group were significantly elevated in comparison to the control, AC, AgNP, AC + AgNP, and L-DOPA + 6-OHDA groups (*p* < 0.001). In comparison to the 6-OHDA group, TNF-α levels were markedly diminished in the AC + 6-OHDA, AgNP + 6-OHDA, and AC + AgNP + 6-OHDA groups (*p* < 0.001), while no significant difference was noted in the L-DOPA + 6-OHDA group (*p* > 0.05). In addition, IL-1β concentrations were significantly elevated in the 6-OHDA group in comparison with the control, AC, AgNP, AC + AgNP, and L-DOPA + 6-OHDA groups (*p* < 0.001). Levels of IL-1β were found to be significantly reduced in the L-DOPA + 6-OHDA, AC + 6-OHDA, AgNP + 6-OHDA, and AC + AgNP + 6-OHDA groups in comparison to the 6-OHDA group (*p* < 0.001). The results of the serum TNF-α and IL-1β analysis for the experimental groups are illustrated in Fig. 2B.

#### LC–MS/MS Analysis Results

In the LC–MS/MS analyses, the transitions m/z 153.8 → 90.9/136.8 for dopamine and m/z 145.9 → 86.9/60.1 for acetylcholine were validated, and the consistency of retention times across all groups supported the reliability of the analytical method. The observation that dopamine and acetylcholine levels were comparable or showed an increasing trend in the control, AC, AgNPs, and AC + AgNPs groups indicates that these treatments did not exert toxic effects on the dopaminergic or cholinergic systems; rather, they may play a modulatory role. In contrast, a marked reduction in both neurotransmitters was observed in the 6-OHDA group, which is consistent with Parkinson-like neurochemical pathology characterized by disruption of the dopamine–acetylcholine balance.

In the treatment groups (L-DOPA + 6-OHDA, AC + 6-OHDA, AgNPs + 6-OHDA, and AC + AgNPs + 6-OHDA), dopamine levels were partially increased compared with the 6-OHDA group, whereas improvements in acetylcholine levels were more limited and heterogeneous. In the combination treatment group, the increasing trend in dopamine levels was preserved, while changes in acetylcholine levels remained relatively modest. Although MRM peak areas and quantitative plots supported these trends, statistical analyses did not reveal significant differences between groups (*p* > 0.05). This finding indicates that the observed effects are at the trend level and should be confirmed in studies with larger sample sizes. Furthermore, the ability of AgNPs to cross the blood–brain barrier [38] may represent a potential mechanism underlying the neurochemical changes observed, particularly in the S-AgNPs-containing groups.

**Fig. 3:**
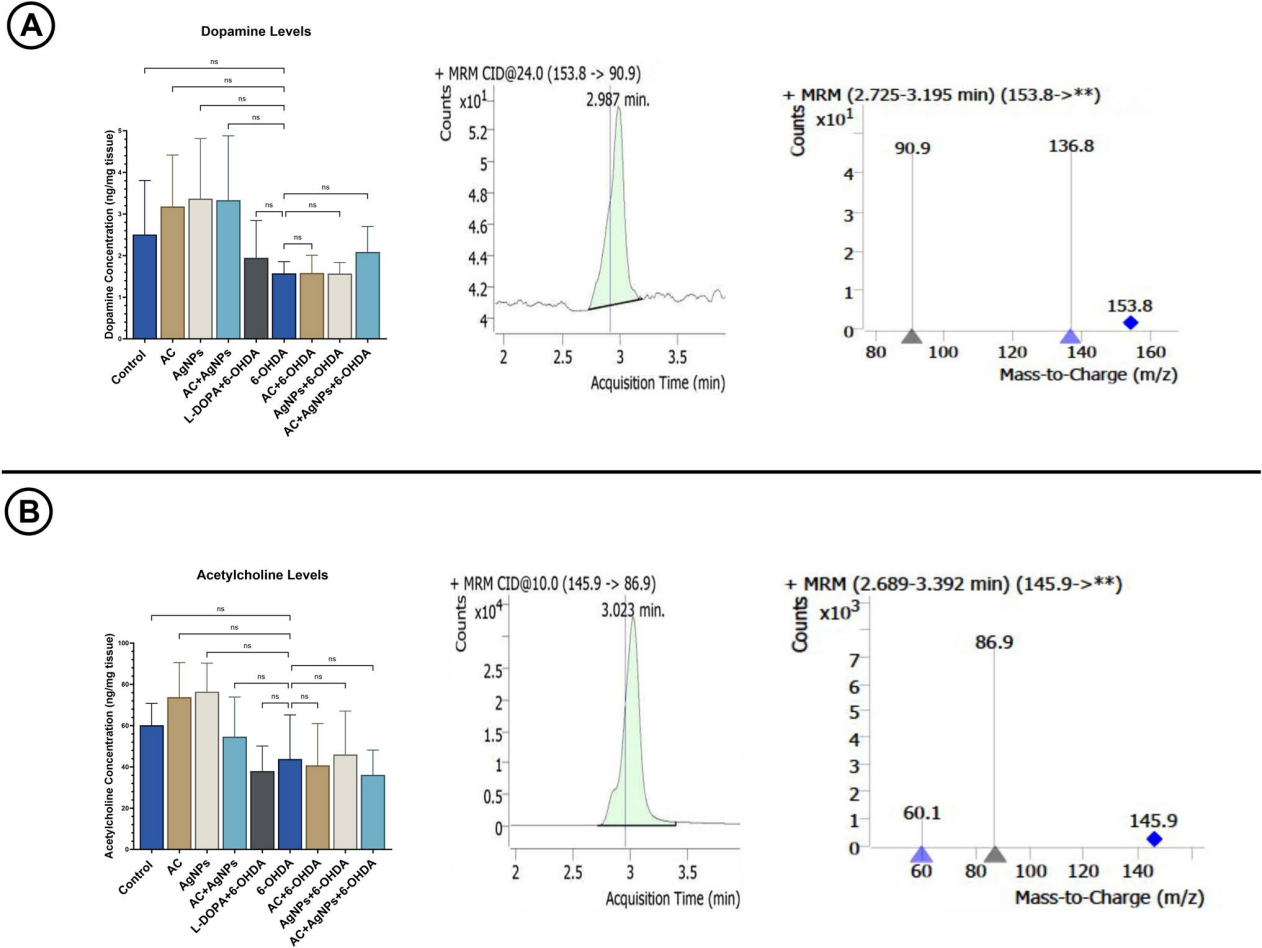
Overview of the changes in brain dopamine (**A**) and acetylcholine (**B**) levels among experimental groups as determined by LC–MS/MS MRM analysis. Statistical significance is indicated by ns (ns, *p* > 0.05)

All evaluations of dopamine and acetylcholine concentrations were based on chromatographic profiles of fragmentation products detected in MRM mode, corresponding mass spectra, and quantitative graphs. The relevant data are presented in Fig. 3A (dopamine) and Fig. 3B (acetylcholine).

#### Histopathological Analysis Results

Histopathological examination revealed that neuronal architecture in the SNpc region was preserved in the control, AC, and AgNP groups, with no evidence of degenerative changes or glial activation. In the L-DOPA group, neuronal loss was partially reduced, although some morphological alterations were still evident. In contrast, the 6-OHDA group exhibited marked neuronal loss and pronounced degenerative changes.

**Fig. 4:**
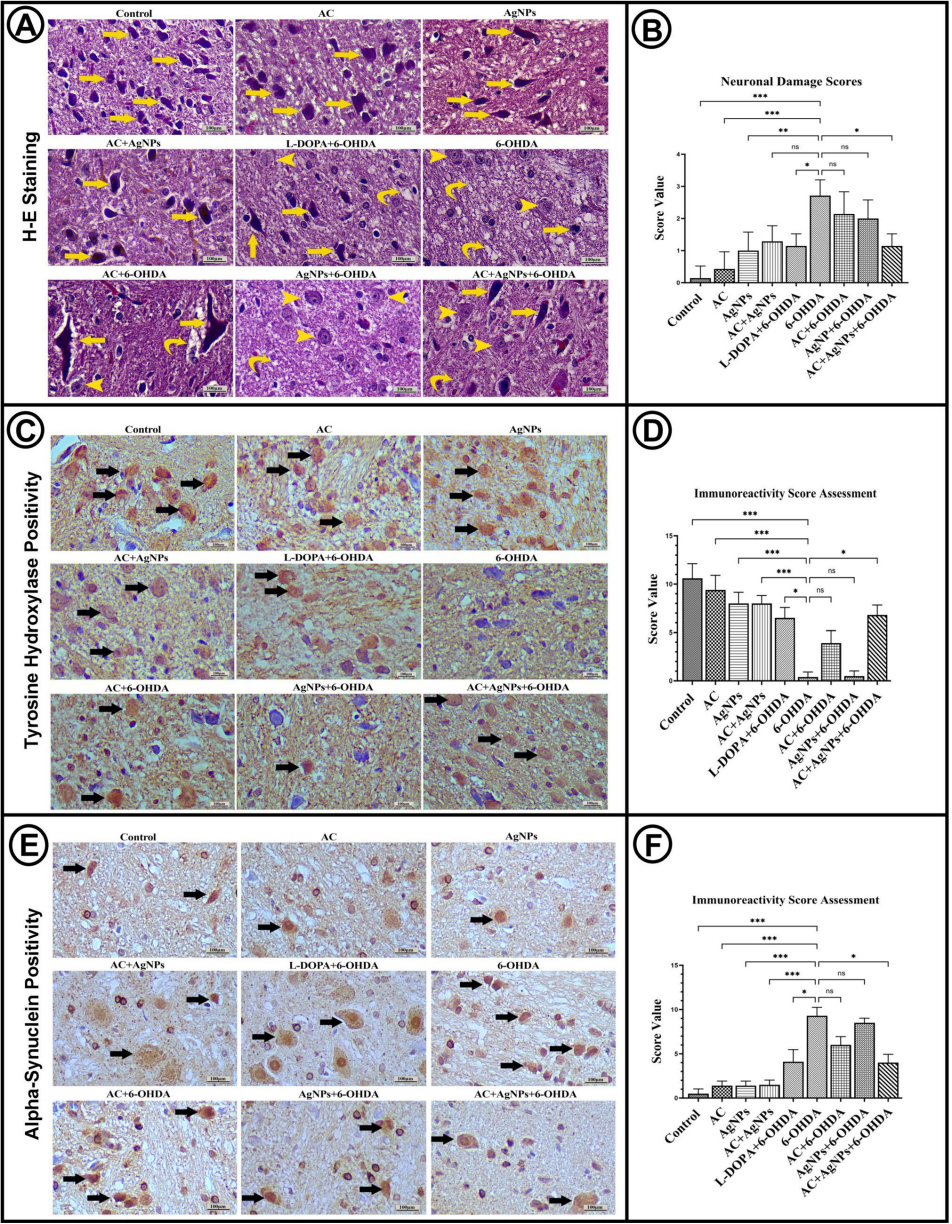
**A** Histopathological analysis of brain sections from all groups (H&E staining). **Yellow arrow**: normal dopaminergic neurons; **yellow arrowhead**: degenerative neurons; **yellow slanted arrow**: cytoplasmic vacuoles. **B** Comparison of neuronal damage scores among groups. **C**,** E** Immunohistochemical detection of tyrosine hydroxylase (TH) and α-synuclein in substantia nigra tissue sections. **Black arrow**: indicates positive cells. **D**,** F**: Group-wise IRS scoring of immunopositivity for tyrosine hydroxylase (TH) and α-synuclein. Statistical significance is indicated by asterisks (*p < 0.05, **p < 0.01, ***p < 0.001, and ns: *p* > 0.05). Scale bars: 100 µm. Staining performed using the HRP-DAB method

In the treatment groups, varying degrees of neuronal preservation were observed. Minimal degenerative changes were noted in the AC + 6-OHDA group, increased neuronal density was observed in the AgNP + 6-OHDA group, and the highest level of histological protection was evident in the AC + AgNP + 6-OHDA group. Overall, while 6-OHDA induced pronounced pathological alterations, AC, AgNP, and their combination markedly attenuated neuronal damage (Fig. 4A).

#### Neuronal Damage Scoring

Neuronal damage was evaluated on hematoxylin and eosin–stained tissue sections using samples obtained from five non-overlapping fields per section. Damage was defined based on features such as cytoplasmic shrinkage and nuclear pyknosis. Assessments were performed in a double-blinded manner, and the proportion of damaged neurons relative to the total neuronal count was calculated as previously described [35]. Histopathological analyses demonstrated pronounced neuronal degeneration in the 6-OHDA group, characterized by cytoplasmic shrinkage, pyknosis, and vacuolization. The neuronal damage score in the 6-OHDA group was significantly higher than that of the control and AC groups (*p* < 0.001), the AgNP group (*p* < 0.01), and the L-DOPA + 6-OHDA group (*p* < 0.05). While AC, AgNP, and combination treatments partially preserved neuronal structure, the AC + AgNP + 6-OHDA group exhibited a significant reduction in damage score compared with the 6-OHDA group (*p* < 0.05), with substantial preservation of the morphological integrity of substantia nigra neurons (Fig. 4B).

### Immunohistochemical Analyses

Immunohistochemical examination demonstrated dense and morphologically preserved TH-positive dopaminergic neurons with homogeneous staining in the SNpc region of the control, AC, AgNP, and AC + AgNP groups. In the 6-OHDA group, TH immunoreactivity was markedly reduced, accompanied by extensive neuronal loss and morphological deterioration. Partial restoration of TH positivity was observed in the L-DOPA + 6-OHDA, AgNP + 6-OHDA, and AC + 6-OHDA groups, whereas the highest level of TH immunoreactivity and substantial preservation of neuronal morphology were evident in the AC + AgNP + 6-OHDA group (Fig. 4C).

Tyrosine hydroxylase (TH) expression was evaluated using the immunoreactivity scoring (IRS) system. TH positivity was comparable among the control, AC, AgNP, and AC + AgNP groups and was significantly higher than that observed in the 6-OHDA group. TH immunoreactivity was significantly increased in the L-DOPA + 6-OHDA and AC + AgNP + 6-OHDA groups compared with the 6-OHDA group, whereas the increases observed in the AC + 6-OHDA and AgNP + 6-OHDA groups did not reach statistical significance (Fig. 4D).

Alpha-synuclein (α-syn) expression was minimal and homogeneous in the control, AC, AgNP, and AC + AgNP groups, with no inclusion formation observed. In contrast, the 6-OHDA group exhibited a marked increase in α-syn expression, accompanied by the formation of aggregate- or inclusion-like structures. Partial reductions were observed in the L-DOPA + 6-OHDA and AC + 6-OHDA groups, a moderate reduction was evident in the AgNP + 6-OHDA group, and minimal expression with an absence of pathological structures was observed in the AC + AgNP + 6-OHDA group (Fig. 4E).

α-syn expression was further quantified using the IRS system. α-syn immunoreactivity was low and comparable in the control, AC, AgNP, and AC + AgNP groups. A significant increase was observed in the 6-OHDA group (*p* < 0.001), which was partially reduced in the L-DOPA + 6-OHDA group (*p* < 0.05). No significant differences were detected in the AC + 6-OHDA and AgNP + 6-OHDA groups (*p* > 0.05), whereas a significant reduction in α-syn immunoreactivity was observed in the AC + AgNP + 6-OHDA group (*p* < 0.05) (Fig. 4F).

#### Western Blot Analysis Results

**Fig. 5:**
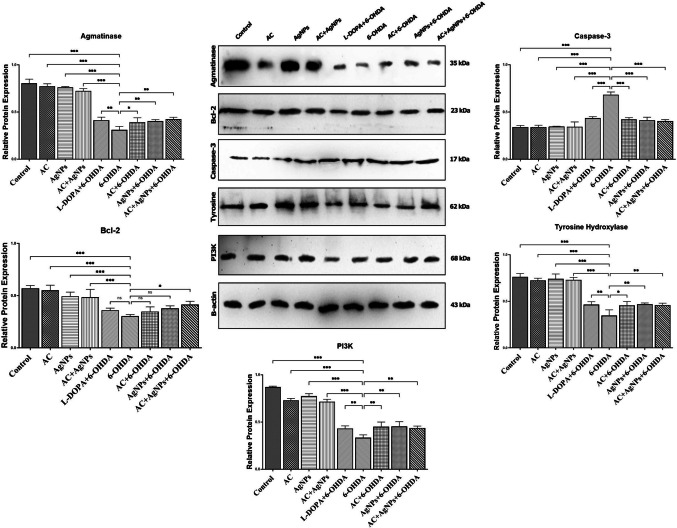
Relative protein expression levels of Agmatinase, Bcl-2, Caspase-3, tyrosine hydroxylase, and PI3K in brain tissues from PD-induced rats. Statistical analysis was performed using one-way ANOVA followed by Tukey’s post hoc test. ns, not significant; *p < 0.05; **p < 0.01; ***p < 0.001, and ns: *p* > 0.05. Error bars represent the standard deviation from at least three independent experiments

Western blot analysis demonstrated that agmatinase levels were significantly reduced in the 6-OHDA group compared with the control, AC, AgNP, and AC + AgNP groups (*p* < 0.001), and were also lower than those observed in the L-DOPA + 6-OHDA, AgNP + 6-OHDA, and AC + AgNP + 6-OHDA groups (*p* < 0.01). In addition, agmatinase expression was significantly decreased in the 6-OHDA group compared with the AC + 6-OHDA group (*p* < 0.05). Bcl-2 levels were significantly reduced in the 6-OHDA group compared with the control, AC, AgNP, and AC + AgNP groups (*p* < 0.001). No significant differences were detected between the 6-OHDA group and the L-DOPA + 6-OHDA, AC + 6-OHDA, or AgNP + 6-OHDA groups (*p* > 0.05); however, a significant difference was observed when compared with the AC + AgNP + 6-OHDA group (*p* < 0.05). Caspase-3 levels were significantly elevated in the 6-OHDA group compared with all other groups (*p* < 0.001). Tyrosine hydroxylase (TH) levels were significantly decreased in the 6-OHDA group compared with the control, AC, AgNP, and AC + AgNP groups (*p* < 0.001). When compared with the L-DOPA + 6-OHDA, AgNP + 6-OHDA, and AC + AgNP + 6-OHDA groups, the differences remained significant (*p* < 0.01), and a significant difference was also observed relative to the AC + 6-OHDA group (*p* < 0.05). PI3K levels were significantly reduced in the 6-OHDA group compared with the control, AC, AgNP, and AC + AgNP groups (*p* < 0.001). Comparisons with all other treatment groups revealed significant differences at the *p* < 0.01 level. The protein expression results for all experimental groups are presented in Fig. 5.

## Discussion

Parkinson’s disease (PD) represents one of the most prevalent neurodegenerative conditions globally, ranking second only to Alzheimer’s disease in frequency [39, 40]. The disease is defined by a gradual degeneration of dopaminergic neurons within the substantia nigra pars compacta (SNpc), accompanied by abnormal aggregation of α-synuclein (α-syn), which impairs mitochondrial function and lysosomal degradation, and induces endoplasmic reticulum (ER) stress [41–43]. The resulting dopamine deficiency disrupts basal ganglia function, manifesting clinically as bradykinesia, rigidity, tremor, and postural instability [44]. Histopathologically, Lewy bodies containing α-syn are hallmarks of dopaminergic neuronal degeneration. Despite advances in understanding PD pathogenesis, no disease-modifying therapies exist, and current treatments primarily provide symptomatic relief [45]. PD involves complex and multifactorial mechanisms, including oxidative stress, neuroinflammation, α-syn accumulation, mitochondrial dysfunction, and impaired survival signaling. Therapeutic strategies targeting single pathways have been insufficient, highlighting the need for multitargeted approaches capable of modulating several pathological mechanisms concurrently [46]. Nanoparticle-based drug delivery represents a promising strategy to achieve effective central nervous system (CNS) bioavailability, particularly across the blood–brain barrier (BBB) [47]. Silver nanoparticles (AgNPs) possess antioxidant and anti-inflammatory properties, and citrate-coated AgNPs enhance biocompatibility, prevent aggregation, prolong circulation, and facilitate BBB penetration [48, 49]. Combining nanoparticles with natural compounds, such as *Antrodia cinnamomea* (AC), may produce synergistic pharmacological effects, improving therapeutic outcomes [50, 51].

In the present study, the neuroprotective effects of AC, alone and conjugated with citrate-coated AgNPs, were evaluated in 6-hydroxydopamine (6-OHDA)-induced in vitro and in vivo PD models. 6-OHDA selectively targets dopaminergic neurons due to structural similarity to dopamine, allowing reliable induction of SNpc neurodegeneration [52, 53]. SH-SY5Y cells differentiated with retinoic acid provide a dopaminergic-like neuronal phenotype suitable for assessing neuroprotective interventions, activating MAPK/ERK and PI3K signaling pathways essential for cell survival and neuronal development [54, 55]. Exposure to 6-OHDA significantly reduced cell viability compared to controls (*p* < 0.001), consistent with oxidative stress-mediated apoptosis reported in previous studies [56]. AC treatment significantly improved cell viability, suggesting suppression of oxidative stress and neuroinflammatory pathways [57]. AC + AgNP treatment also increased cell viability (*p* < 0.05), albeit to a lesser degree. In contrast, monotherapies with AgNP, L-DOPA + 6-OHDA, AC + 6-OHDA, or AgNP + 6-OHDA did not produce significant cytoprotective effects, consistent with reports of L-DOPA’s limited antioxidant capacity and possible pro-oxidant effects under certain conditions [58, 59]. AC-AgNP combinations at 25–50 μM enhanced viability, indicating improved intracellular delivery and retention of AC via the nanoparticle carrier system, supporting previous findings that nanocarriers increase the bioavailability and efficacy of natural compounds [60].

Previous studies using 6-OHDA in SH-SY5Y cells have demonstrated that reduced cell viability is closely associated with increased oxidative stress, activation of apoptotic pathways, and suppression of pro-survival signaling, such as the PI3K/Akt pathway [61–63]. Consistent with these reports, our in vivo results showing decreased MDA levels, increased SOD and GSH activities, reduced Caspase-3 expression, and increased Bcl-2 levels suggest that the cytoprotective effects observed in SH-SY5Y cells are likely mediated through similar molecular mechanisms. Behavioral assessments in unilateral 6-OHDA-lesioned rats revealed pronounced motor deficits, including reduced locomotion, increased apomorphine-induced rotations, and forelimb asymmetry in the cylinder test [17, 64, 65]. AC + AgNP treatment significantly improved motor performance, reducing rotational behavior and preserving bilateral forelimb use (*p* < 0.05–0.001), consistent with preservation of nigrostriatal dopaminergic integrity. This aligns with literature demonstrating that multitargeted antioxidant and neuroprotective strategies can mitigate motor deficits in toxin-induced PD models [66, 67].

Systemic inflammation, as indicated by elevated serum TNF-α and IL-1β, was observed in 6-OHDA rats [68, 69]. AC, AgNP, and particularly AC + AgNP treatments reduced these cytokine levels, highlighting modulation of neuroinflammatory pathways. L-DOPA did not significantly alter inflammatory markers, reflecting its primarily symptomatic dopaminergic replacement role [70]. Oxidative stress analysis revealed increased MDA and decreased GSH and SOD in 6-OHDA-treated rats [71, 72]. AC and AC + AgNP significantly restored redox balance (*p* < 0.05), supporting their role in enhancing endogenous antioxidant defenses [73, 74]. L-DOPA had a limited effect, consistent with its symptomatic mechanism [75].

Histopathological and immunohistochemical assessments corroborated these findings. In the control, AC, AgNP, and AC + AgNP groups, SNpc neuronal morphology was preserved, TH immunoreactivity was strong, and α-syn expression remained physiological [76–78]. 6-OHDA induced severe dopaminergic neuron loss, reduced TH staining, Lewy body-like α-syn aggregation, and pronounced inflammation [76]. L-DOPA partially improved TH immunoreactivity but did not prevent α-syn accumulation [79–81]. AC or AgNP monotherapies attenuated inflammation and α-syn positivity but had limited effect on TH-positive cell density. The AC + AgNP combination produced the most pronounced neuroprotection, preserving morphology, reducing inflammation, maintaining TH expression, and preventing pathological α-syn aggregation [82, 83]. At the molecular level, 6-OHDA decreased PI3K, Bcl-2, agmatinase, and TH, while increasing Caspase-3, indicating suppression of survival pathways and activation of apoptosis [84–86]. AC + AgNP restored PI3K/Akt signaling, increased Bcl-2 expression, suppressed Caspase-3 activity, and partially restored agmatinase activity, confirming synergistic neuroprotective effects [87]. Dopamine levels, measured by MRM, were partially restored by AC and AgNP and maximally by AC + AgNP, suggesting functional preservation of the dopaminergic system [88].

Taken together, these findings suggest that the neuroprotective effects of AC-loaded AgNPs are mediated through the coordinated modulation of oxidative stress, neuroinflammatory responses, and apoptosis-related signaling pathways, ultimately preserving dopaminergic neuronal integrity and improving motor function in the 6-OHDA-induced Parkinsonian model. Importantly, the improvement observed in behavioral motor tests was consistent with the biochemical restoration of antioxidant defenses, preservation of dopaminergic neurons in the substantia nigra, and normalization of apoptosis-related signaling pathways.

In the literature, studies using the 6-OHDA rodent model have demonstrated that dopaminergic degeneration in the nigrostriatal pathway occurs progressively during the early post-lesion period following toxin administration. For example, unilateral injection of 6-OHDA into the medial forebrain bundle results in progressive dopaminergic neuron loss in the substantia nigra pars compacta during the first 1–3 weeks after lesion induction, before reaching a relatively stable phase, as demonstrated by stereological quantification of tyrosine hydroxylase (TH)-positive neurons at different post-lesion time points [89, 90]. However, unlike the slow and chronic progression observed in human Parkinson’s disease, the 6-OHDA model typically produces a rapid toxin-induced lesion in which motor deficits and neurochemical depletion develop within days to weeks and subsequently plateau rather than continuously worsen over extended periods. Biological factors such as natural aging and other physiological modifiers may also influence the severity and behavioral expression of 6-OHDA-induced Parkinsonian features [91].

In the present study, we employed the 6-OHDA model, which induces unilateral nigrostriatal dopaminergic neuron loss and asymmetric motor deficits; injections into the substantia nigra or striatum produce rapid neuronal degeneration and progressive retrograde disruption of the nigrostriatal pathway in rodents [92]. In comparison, the MPTP model causes bilateral dopaminergic degeneration, replicating many motor features of Parkinson’s disease, and is widely used to investigate neurotoxic pathways in mice [93, 94]. The LRRK2 genetic model, on the other hand, results in a slower, progressive dopaminergic deficit, reflecting familial forms of Parkinson’s disease and enabling studies of long-term pathogenic mechanisms [95]. Application of the same AC-loaded AgNP treatment across these models could be expected to yield differential outcomes. In MPTP-treated animals, the systemic and bilateral nature of degeneration may require higher or repeated doses to achieve comparable neuroprotection. In LRRK2 models, the slower progression may allow modulation of long-term neuronal survival and behavioral performance, although acute effects might be less pronounced than in the 6-OHDA model.

Notwithstanding these promising findings, several limitations of the present work should be acknowledged. First, the 6-hydroxydopamine (6-OHDA) model selectively reproduces nigrostriatal dopaminergic degeneration but does not fully replicate the progressive, multifactorial pathology of human Parkinson’s disease, including Lewy body formation and chronic α-synuclein accumulation [96]. Second, while citrate-coated silver nanoparticles (AgNPs) showed neuroprotective potential, their biological effects may vary with particle size, coating, and dosage; higher doses or different coatings may be neurotoxic, necessitating further studies on long-term safety, pharmacokinetics, and optimal dosing [97]. Third, the bioactive composition of *Antrodia cinnamomea* (AC) extracts may differ with cultivation, extraction methods, and concentration of active compounds, potentially affecting reproducibility [98]. Fourth, experiments were conducted exclusively in male rats; sex-related differences in neurodegeneration, neuroinflammation, and therapeutic outcomes warrant inclusion of female animals in future studies to assess potential sex-dependent effects [96, 99]. Future studies are needed to perform detailed molecular analyses in SH-SY5Y cells to directly confirm the mechanistic parallels observed in the in vivo Parkinsonian model. Fifth, L-DOPA does not mitigate oxidative stress or inflammation and may have long-term adverse effects, which should be considered when evaluating therapeutic outcomes [100]. Finally, this study focused on short-term neuroprotective effects; long-term investigations using chronic or progressive PD models, combined with detailed toxicological and pharmacokinetic analyses, are required to fully validate the translational potential of AC-conjugated AgNPs.

In conclusion, citrate-coated *Antrodia cinnamomea* (AC)-conjugated silver nanoparticles (AC + AgNPs) provide multitargeted neuroprotection in a 6-OHDA-induced Parkinson’s disease model through modulation of oxidative stress, neuroinflammation, and apoptotic pathways. Behavioral assessments demonstrated that AC + AgNP treatment reduced apomorphine-induced rotational asymmetry, indicating improved dopaminergic function. Biochemical analyses revealed attenuation of oxidative stress, with decreased lipid peroxidation and restored antioxidant defenses. Histopathological and immunohistochemical evaluations confirmed preservation of neuronal morphology in the substantia nigra, maintenance of dopaminergic neuronal integrity, restoration of tyrosine hydroxylase expression, and suppression of pathological α-synuclein accumulation. Western blot analyses further indicated modulation of apoptotic and survival pathways, supporting enhanced neuroprotection.

## Supplementary Information

Below is the link to the electronic supplementary material.

ESM 1 (DOCX 1.80 MB)

ESM 2 (PDF 420 KB)

## Data Availability

The datasets generated during the current study are available from the corresponding author on reasonable request.

## Declarations

### Use of Artificial Intelligence in Manuscript Preparation

During the preparation of this manuscript, ChatGPT 3.5 was employed solely to assist with grammatical accuracy and spelling consistency.
