# Supplementary material for: Modulation of Oxidative Stress and Apoptosis by Antrodia cinnamomea–Loaded Citrate-Stabilized Silver Nanoparticles in Experimental Parkinsonism
Source: Mol Neurobiol. 2026 Apr 21;63(1):576. doi: 10.1007/s12035-026-05853-5 (PMC13099803; doi:10.1007/s12035-026-05853-5)
Supplement: Supplementary file 1 — (DOCX 1.80 MB) [file 12035_2026_5853_MOESM1_ESM.docx]

***Supplements:***

**Modulation of Oxidative Stress and Apoptosis by Antrodia cinnamomea–Loaded Citrate-Stabilized Silver Nanoparticles in Experimental Parkinsonism**

Deniz Tekiner,VMD, PhD ^1 a *^, Semin Gedikli,VMD PhD ^1 a^, Volkan Gelen, VMD, PhD^2 b^ , Cemil Bayram VMD, PhD ^3 c^, Adem Kara VMD, PhD ^4 d^

^1^ Department of Histology and Embryology, Faculty of Veterinary, Atatürk University, Erzurum/ Türkiye,

^2^ Department of Physiology, Faculty of Veterinary, Kafkas University, Kars/ Türkiye,

^3^ Department of Pharmacology and Toxicology, Faculty of Veterinary, Atatürk University, Erzurum/ Türkiye,

^4^ Department of Molecular Biology and Genetics, Faculty of Science, Erzurum Technical University, Erzurum/ Türkiye.

***Authors’ ORCID iDs***

1:0000-0002-1950-1708

2:0000-0001-8238-7226

3:0000-0002-5091-1262

4:0000-0001-8940-8560

5:0000-0002-5766-6116

*** Corresponding author:**

1. Dr. Deniz TEKİNER

Department of Histology and Embryology, Faculty of Veterinary

Atatürk University/Türkiye

deniz.tekiner@atauni.edu.tr

0000-0002-1950-1708

**Supplementary Methods**

**Preparation and Analytical Evaluation of Citrate-Coated Silver Nanoparticles (AgNPs)**

Silver nanoparticles (AgNPs) were synthesized using a citrate-mediated reduction method adapted from well-established protocols (1). Briefly, 0.0167 g of silver nitrate (AgNO₃) was dissolved in 100 mL of distilled water. Separately, 0.020 g of sodium citrate (Na₃C₆H₅O₇) was dissolved in 20 mL of distilled water. The silver nitrate solution was heated to boiling while stirring. Upon reaching the boiling point, 5 mL of the sodium citrate solution was added dropwise, and the mixture was continuously boiled with magnetic stirring for approximately 1 hour. The color change from light yellow to dark brown indicated the reduction of Ag⁺ to Ag⁰ and the formation of AgNPs. Formation of nanoparticles was confirmed by ultraviolet-visible (UV–Vis) spectroscopy, showing a characteristic absorption peak at approximately 427 nm. The solution was allowed to cool to room temperature and subsequently stored at 4 °C in the dark until further use.

**Characterization of Citrate-Coated AgNPs**

Fourier transform infrared (FT-IR) spectroscopy was employed to determine the functional groups present on the nanoparticles. FT-IR spectra were obtained using an FT-IR spectrophotometer operating within the 400–4000 cm⁻¹ range **(Supplement 1A)**. The AgNPs were further analyzed through several complementary techniques. Their optical characteristics were evaluated using ultraviolet–visible (UV-Vis) spectroscopy with a Perkin Elmer Lambda 35 spectrophotometer, spanning wavelengths from 200 to 800 nm **(Supplement 1B)**. Additionally, transmission electron microscopy (TEM) was performed using a Hitachi HighTech-7700 instrument, providing high-resolution images that allowed detailed morphological examination of the nanoparticles **(Supplement 1C)**.

**
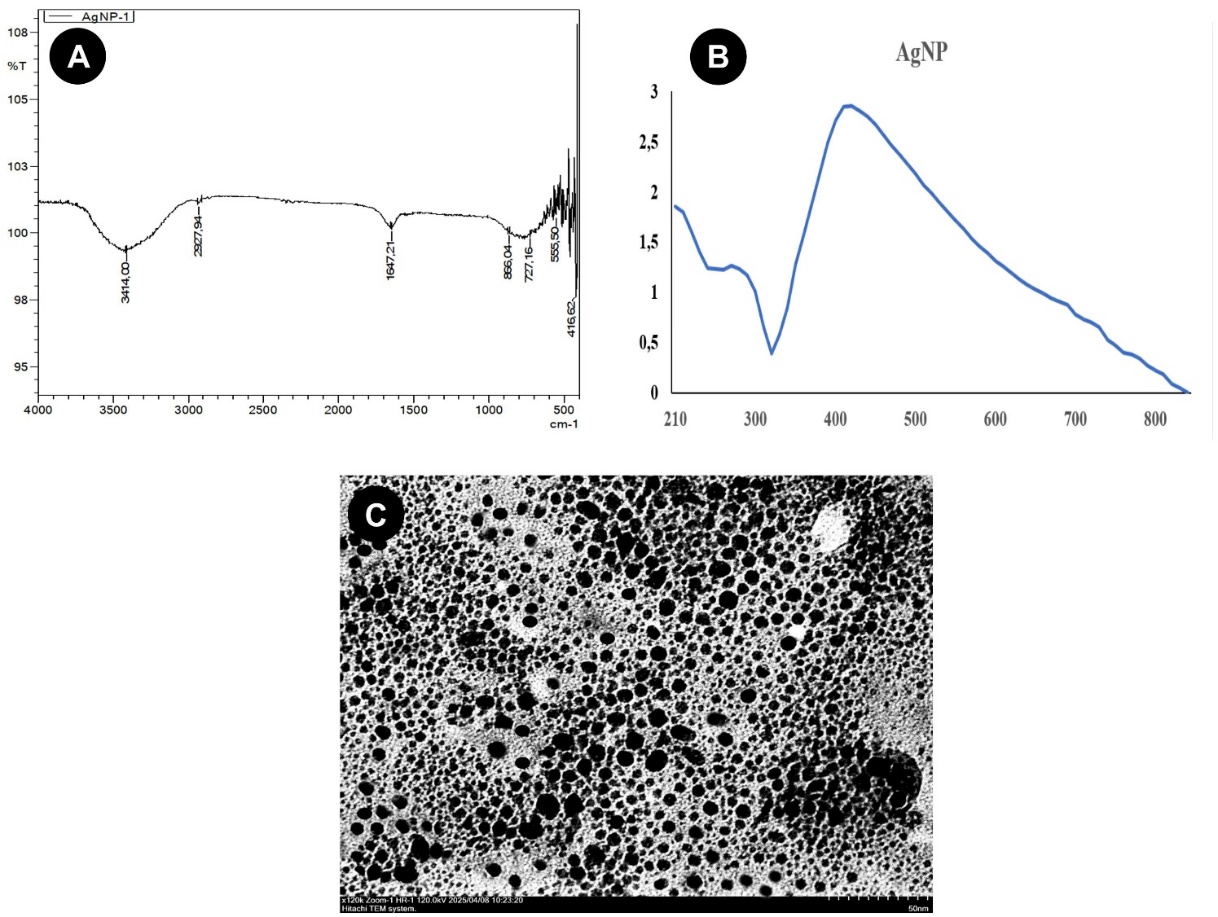
**

**Supplement 1.** Characterization of Silver Nanoparticles (AgNPs) (A); FT-IR Spectrum of Citrate-Coated Silver Nanoparticles (AgNPs), (B); UV Spectrum of Citrate-Coated Silver Nanoparticles (AgNPs), (C); Transmission Electron Microscopy (TEM) Spectrum of Silver Nanoparticles (AgNPs).

**Experimental Design**

The rats were divided into nine groups with seven animals each for the experimental applications, and treatment with Antrodia cinnamomea (AC) was initiated. The animals' body weights were recorded daily, and the administration of AgNPs (1 mg/kg) (2) and/or AC (100 mg/kg) (3) was carried out over 14 days **(see Supplement 5 for detailed experimental design)**. On the 14th day, PD was induced by unilaterally injecting 6-OHDA into the substantia nigra. According to stereotaxic coordinates relative to Bregma, the injection site was set at anteroposterior (AP) -5.5 mm, mediolateral (ML) +2.0 mm, and dorsoventral (DV) -8 mm, based on the rat brain atlas of Paxinos and Watson (4). The coordinates for the injection sites are presented **(Supplement 2A)**. Drilling of the burr hole at the marked coordinates **(Supplement 2B)**. All experimental groups, except for the healthy control group, received stereotaxic injections of 6-hydroxydopamine (6-OHDA) into the substantia nigra pars compacta (SNpc). The neurotoxin was dissolved in 0.1% ascorbic acid in physiological saline to prevent oxidation and prepared at a concentration of 8 μg/μL. A total volume of 4 μL of this solution (total dose: 32 μg per animal) was administered stereotaxically using a Hamilton syringe at a constant infusion rate over 2 minutes. In stereotaxic neurotoxin models, the lesion is typically standardized based on the absolute amount of toxin delivered directly to the target brain region rather than body weight; therefore, the same dose was administered to all animals. Based on the body weights of the animals used in this study, the administered dose corresponds to approximately 120–140 μg/kg body weight. The use of 6‑OHDA in stereotaxic models is a well‑established methodology in preclinical Parkinson’s disease research. In these protocols, the toxin is typically administered at a defined absolute amount directly into the target region rather than scaled to body weight, as the important factor is the local effect on dopaminergic neurons rather than systemic exposure. This approach is consistent with previously reported protocols demonstrating reliable dopaminergic lesion induction following stereotaxic 6-OHDA administration to the SNpc (1,5). The injection administration process is presented **(Supplement 2C)**. Behavioral assessments, including the locomotor activity test, cylinder test, and apomorphine-induced rotation test, were performed 48 hours after the injection.

At the end of the experiment, blood samples were collected from all animals via the left cardiac ventricle under isoflurane inhalation anesthesia. Subsequently, the animals were euthanized, and the brain tissues were carefully removed. The macroscopic appearance of the brain tissue from the group subjected to 6-OHDA injection. The arrow indicates the injection site **(Supplement 2G)**. The harvested brain tissues were then fixed in 4% formaldehyde solution for histopathological analysis.The arrow indicates the injection site **(Supplement 2H)**. All histopathological and immunohistochemical stainings were performed on coronal brain sections encompassing the substantia nigra **(Supplement 2I)**, obtained using a microtome.

The number of animals per group (n = 7) was determined in accordance with sample sizes reported in previous studies employing comparable 6-OHDA–induced PD models (1,6). A post hoc power analysis conducted using G*Power 3.1 (α = 0.05), based on the effect sizes obtained from our primary outcome measures, demonstrated a statistical power (1–β) exceeding 0.80 for detecting medium-to-large effect sizes (Cohen’s f ≥ 0.4) in one-way ANOVA analyses

**Behavior tests**

**1-Cylinder Test**

The cylinder test is a sensitive behavioral method widely used in experimental Parkinson’s disease models to assess the impact of unilateral dopaminergic lesions on motor asymmetry. Quantifying lateralization in forelimb use, it enables objective evaluation of both disease-related motor deficits and treatment-induced functional improvement (7,8).

Two days later post-injection, the cylinder test was performed in which each animal was individually placed in a transparent Plexiglas cylinder (20 cm diameter, 30 cm height). During spontaneous exploratory behavior, animals rear on their hind limbs and contact the inner wall using the right, left, or both forelimbs. All behaviors were recorded for 60 minutes and analyzed using slow-motion video review **(Supplement 2D)**.

Forelimb contacts were categorized as right, left, or bilateral. A decrease in contralateral forelimb use was considered an indicator of dopaminergic dysfunction. For each animal, the percentage of contralateral forelimb use was calculated and statistically compared across groups (9).

Results were expressed as percentages; the total number of forelimb contacts served as the reference for calculating category-specific percentages. To ensure unbiased evaluation, observers analyzing the videos were blinded to the treatment groups.

**2-Evaluation of Apomorphine-Induced Rotational (AIR) Behavior**

The apomorphine-induced rotational behavior test is one of the most commonly used behavioral assays to assess dopaminergic imbalance in experimental models of Parkinson’s disease (10). In animals with unilateral nigrostriatal lesions, administration of apomorphine—a non-selective dopamine agonist with high receptor affinity—reveals increased postsynaptic receptor sensitivity due to dopamine depletion on the lesioned side, resulting in repeated contralateral turning behavior (1,9). This test was performed exclusively in the 6-OHDA-lesioned groups to confirm unilateral dopaminergic denervation; non-lesioned control groups were not subjected to the assessment.

Following the cylinder test, animals were placed in an open-top observation chamber (280 mm in diameter, 380 mm in height) and allowed a 5-minute adaptation period before testing. Apomorphine HCl was administered intraperitoneally at a dose of 2.5 mg/kg, and the total number of complete contralateral rotations was recorded over a 30-minute observation period. Behavioral assessments were performed by investigators blinded to group allocation. Contralateral turns were scored as positive (+) and ipsilateral turns as negative (–), with the net rotation score calculated as the difference between these values. This net score served as a behavioral indicator of treatment efficacy **(Supplement 2E).**

The apomorphine-induced rotation test is one of the most commonly used behavioral methods to assess dopaminergic imbalance in experimental models of Parkinson’s disease. In animals with unilateral nigrostriatal lesions, administration of apomorphine—a non-selective dopamine agonist with high receptor affinity—reveals increased postsynaptic receptor sensitivity due to dopamine depletion on the lesioned side, resulting in repeated contralateral turning behavior (9). Following the cylinder test, animals were placed in an open-top observation chamber (280 mm in diameter, 380 mm in height) and allowed a 5-minute adaptation period before testing. Following preparation of the video recording system, apomorphine HCl was administered intraperitoneally at a dose of 2.5 mg/kg. After injection, behaviors were recorded for 30 minutes, and 360° full rotations were quantified during video analysis. Contralateral turns were scored as positive (+) and ipsilateral turns as negative (–), with the net rotation score calculated as the difference between these values. This net score was used as a behavioral indicator of treatment efficacy (11)

**3-Locomotor Activity**

The locomotor activity test was conducted to objectively assess motor impairments in the Parkinson’s disease model. Each animal was individually placed in an open-topped observation chamber (420 × 420 × 420 mm), and spontaneous activities were recorded for 20 minutes using the May Act 508 system. The device automatically measured ambulatory activity, horizontal and vertical movements, stereotypic behavior, resting time, and total distance traveled. At the end of the recording session, the May Act 508 system generated a locomotor activity map for each animal; in this map, blue lines represented horizontal movements, while green lines indicated vertical movements **(Supplement 2F)**. All obtained parameters were evaluated to determine the impact of dopaminergic degeneration on movement patterns and to assess the effectiveness of the applied treatments on motor function (12).


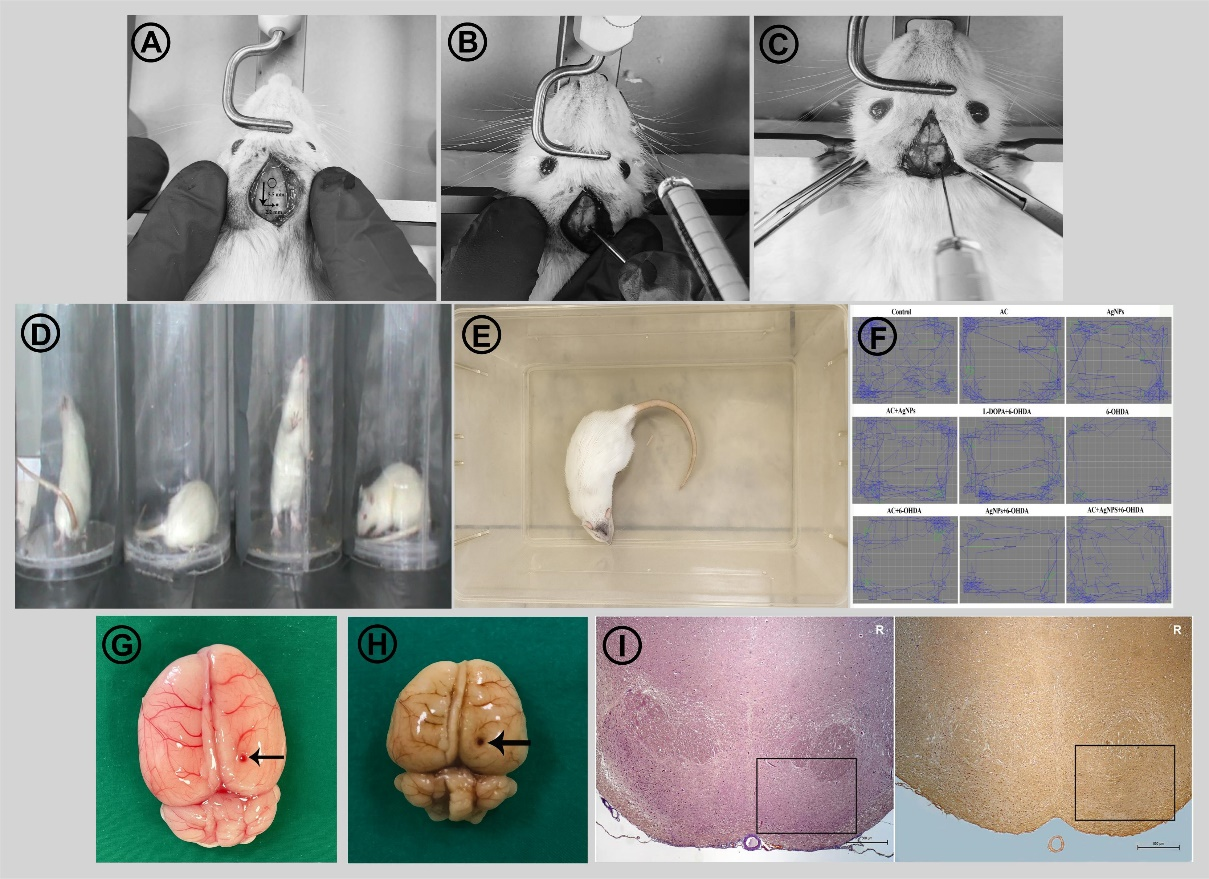


**Supplement 2. A:** The Bregma point is indicated within a circular marker, while the anteroposterior distance of –5.5 mm is shown with a thick directional arrow, and the mediolateral distance of +2.0 mm is denoted by a thin arrow; **B:** Drilling of the burr hole at the marked coordinates; **C:** The injection administration process; **D:** Representative video recording of the Cylinder Test; **E:** Representative image from the apomorphine-induced rotation test; **F:** Locomotor activity map image, in which blue lines represent horizontal movements and green lines indicate vertical movements; **G:** Macroscopic image of the brain from the 6-OHDA–injected group; the arrow indicates the injection site; **H:** Formaldehyde-fixed brain tissue; the arrow indicates the injection site. Images G and H were obtained from the same brain sample; the apparent shrinkage in H is due to tissue processing and dehydration during fixation. **I:** Coronal brain sections including the substantia nigra; the rectangular outline marks the substantia nigra region, where all histopathological and immunohistochemical stainings were performed.

**Cell-Based Models of Parkinson’s Disease: Utilization of the SH-SY5Y Neuroblastoma Cell Line**

Neuroblastoma cells (SH-SY5Y) were retrieved from liquid nitrogen storage at −196 °C and cultured in Dulbecco’s Modified Eagle Medium (DMEM) supplemented with 10% fetal bovine serum (FBS) and 1% penicillin–streptomycin. Once the cells developed appropriate undifferentiated morphological characteristics, they were subcultured and subsequently seeded into 96-well plates at a density of 1,500 cells per well. In accordance with established protocols, cellular differentiation was induced by exposing the cells to 10 µM retinoic acid for 5 days. Following differentiation, neuronal injury was elicited by administering 50 µM 6-hydroxydopamine (6-OHDA). Cell viability was determined using the CVDK-8 colorimetric assay [3-(4,5-dimethylthiazol-2-yl)-2,5-diphenyltetrazolium bromide; Ecotech Biotech, Turkey]. All procedures were conducted in triplicate, with three biological and three technical replicates for each experimental condition. The entire experiment was repeated to validate reproducibility, and the resulting data were analyzed accordingly. Differentiated SH-SY5Y cells were seeded into 96-well plates, and chemical treatments were initiated following a 24-h incubation period. The compounds used, and their respective concentrations, are listed in **Supplement 4**, while representative images of differentiated SH-SY5Y cells are shown in **Supplement 3**. Cell viability was reassessed using the CVDK-8 assay 24 h after treatment. After 2.5 h of incubation with the MTT reagent at 37 °C, absorbance values were recorded at 450 nm using a μQuant (Biotek) microplate spectrophotometer. Cell viability percentages were calculated based on these absorbance measurements.

**
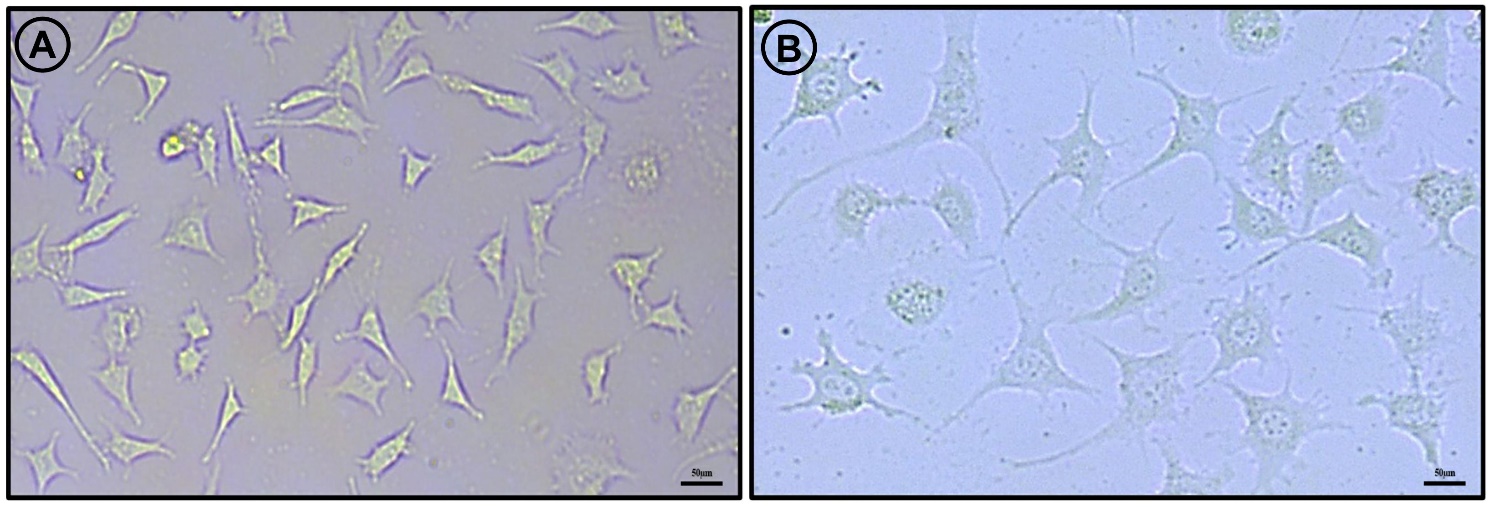
**

**Supplement 3.** SH-SY5Y (Neuroblastoma) cells whose cellular transformation was realized with 10 μM retinoic acid.

Undifferentiated cell morphology (A); Differentiated cell morphology (B). Scale bar 50µm.

**Supplement 4.** Substances and their concentrations applied to cells created in the Parkinson's cellular model

| **Application** | **Cell lines** | **Transformation of cells to neurons via Retinoic acid** | **PD stimulation (hour 0)** | **In vitro concentration**  **(hour 6)** | **In vitro Incubation Duration** |
| --- | --- | --- | --- | --- | --- |
| **X: Antrodia cinnamomea** | SH-SY5Y | 10 μM Retinoic acid for 7 days | 20 μM 6- OHDA | Antrodia cinnamomea (25 and 50 μM concentrations) was applied | 30 hours |
| **Y: Silver**  **Nanoparticle (AgNPs)** | SH-SY5Y | 10 μM Retinoic acid for 7 days | 20 μM 6- OHDA | 5 μg/mL concentrations were applied | 30 hours |
| **Y nanoparticle is attached to X molecule** | SH-SY5Y | 10 μM Retinoic acid for 7 days | 20 μM 6- OHDA | Antrodia cinnamomea (25, 50 μM) + AgNPs (5-μg/mL) were applied | 30 hours |

**Supplement 5.** All experimental groups and applications

| ***Groups*** | ***First 14 days*** | ***At 14. days*** | ***Behavioral test***  ***(16 days)*** | ***End of the experiment (17 days)*** |
| --- | --- | --- | --- | --- |
| ***Control group (n:7)*** | Physiological saline will be given intraperitoneally for 14 days. | - | Behavioral tests will be performed | It will be sacrificed and blood and brain tissues were collected. |
| ***AC group (n:7)*** | Only Antrodia cinnamomea will be administered orally at a dose of 100 mg/kg for 14 days | - | Behavioral tests will be performed | It will be sacrificed and blood and brain tissues were collected. |
| ***AgNPs group (n:7)*** | Citrate-coated silver nanoparticles will be administered orally at a dose of 1 mg/kg for 14 days. | - | Behavioral tests will be performed | It will be sacrificed and blood and brain tissues were collected. |
| ***AC+AgNPs group (n:7)*** | Citrate-coated silver nanoparticle solution at a dose of 1 mg/kg loaded with Antrodia cinnamomea at a dose of 100 mg/kg will be administered orally for 14 days. | - | Behavioral tests will be performed | It will be sacrificed and blood and brain tissues were collected. |
| ***L-DOPA + 6-OHDA group (n:7)*** | Only L-DOPA will be administered orally at a dose of 10 mg/kg for 14 days | 8μg/ μl 6-OHDA will be given intracranially | Behavioral tests will be performed (intraperitoneal administration of apomorphine 2.5 mg/kg) | It will be sacrificed and blood and brain tissues were collected. |
| ***6-OHDA group (n:7)*** | Physiological saline will be given intraperitoneally for 14 days. | 8μg/ μl 6-OHDA will be given intracranially | Behavioral tests will be performed (intraperitoneal administration of apomorphine 2.5 mg/kg) | It will be sacrificed and blood and brain tissues were collected. |
| ***AC+6-OHDA group***  ***(n:7)*** | Antrodia cinnamomea will be given orally at a dose of 100 mg/kg for 14 days. | 8μg/ μl 6-OHDA will be given intracranially | Behavioral tests will be performed | It will be sacrificed and blood and brain tissues were collected. |
| ***AgNPs+6-OHDA group (n:7)*** | Citrate-coated silver nanoparticles will be administered orally at a dose of 1 mg/kg for 14 days. | 8μg/ μl 6-OHDA will be given intracranially | Behavioral tests will be performed (intraperitoneal administration of apomorphine 2.5 mg/kg) | It will be sacrificed and blood and brain tissues were collected. |
| ***AC + AgNPs+6-OHDA group (n:7)*** | Citrate-coated silver nanoparticle solution at a dose of 1 mg/kg loaded with Antrodia cinnamomea at a dose of 100 mg/kg will be administered orally for 14 days. | 8μg/ μl 6-OHDA will be given intracranially | Behavioral tests will be performed (intraperitoneal administration of apomorphine 2.5 mg/kg) | It will be sacrificed and blood and brain tissues were collected. |

**Supplement 6.** The primary antibodies using Immunohistochemical (IHC) and Western blot analysis

| Antibody | Manufacturer | Dilution | Function |
| --- | --- | --- | --- |
| TH (Tyrosine Hydroxylase) | Affinity, AF6113, Affinity Biosciences, China | IHC, 1/100 | TH is a marker that indicates the presence of dopaminergic neurons |
| Alpha-synuclein (α-syn) | Affinity, AF0402, Affinity Biosciences, China | IHC 1/100 | α-syn is a marker that indicates alpha-synuclein accumulation and related neurodegenerative changes |
| Agmatinase | Santa cruz, sc-166414, Santa Cruz Biotechnology, USA | WB, 1/1000 | Agmatinase is a marker that indicates reduced agmatine availability and enhanced neurodegenerative processes in Parkinson’s disease. |
| Bcl-2 | Santa cruz, Sc-7382, Santa Cruz Biotechnology, USA | Wb, 1/1000 | Bcl-2 is a marker that indicates anti-apoptotic activity and cellular survival. |
| Caspase-3 | Santa cruz, sc-65497, Santa Cruz Biotechnology, USA | WB, 1/1000 | Caspase-3 is a marker that indicates apoptosis activation and contributes to dopaminergic neuronal loss in Parkinson’s disease. |
| PI3K | Santa cruz, sc-1637, Santa Cruz Biotechnology, USA | WB1/1000 | PI3K is a marker that reflects the activation of cell-survival signaling pathways and neuroprotective mechanisms in Parkinson’s disease. |
| β-actin | Affinity Biotech. DF70187, Affinity Biosciences, China | WB, 1/1000 | Internal control |
| Secondary antibody | Santa Cruz, sc-2004/sc-2005, Santa Cruz Biotechnology, USA | WB,1/10000 | secondary antibody conjugated to horseradish peroxidase |

**References**

1. Kara H, Tekiner D, Üstündağ H, Bayram C, Şebin SÖ, Özkanlar S, et al. Astaxanthin-Loaded Silver Nanoparticles Mitigate 6-OHDA-Induced Parkinson’s via ER Stress and PI3K/Akt/mTOR Signaling. Mol Neurobiol. 2025 Nov 24;63(1):156. doi:10.1007/s12035-025-05279-5 PubMed PMID: 41276706.

2. Qin G, Tang S, Li S, Lu H, Wang Y, Zhao P, et al. Toxicological evaluation of silver nanoparticles and silver nitrate in rats following 28 days of repeated oral exposure. Environ Toxicol. 2017 Feb;32(2):609–18. doi:10.1002/tox.22263 PubMed PMID: 26996539.

3. Lanza M, Cucinotta L, Casili G, Filippone A, Basilotta R, Capra AP, et al. The Transcription Factor Nrf2 Mediates the Effects of Antrodia camphorata Extract on Neuropathological Changes in a Mouse Model of Parkinson’s Disease. International Journal of Molecular Sciences. 2023 Jan;24(11):11. doi:10.3390/ijms24119250

4. Paxinos G, Watson C, Pennisi M, Topple A. Bregma, lambda and the interaural midpoint in stereotaxic surgery with rats of different sex, strain and weight. J Neurosci Methods. 1985 Apr;13(2):139–43. doi:10.1016/0165-0270(85)90026-3 PubMed PMID: 3889509.

5. Deumens R, Blokland A, Prickaerts J. Modeling Parkinson’s disease in rats: an evaluation of 6-OHDA lesions of the nigrostriatal pathway. Exp Neurol. 2002 Jun;175(2):303–17. doi:10.1006/exnr.2002.7891 PubMed PMID: 12061862.

6. Jovanovic MZ, Stanojevic J, Stevanovic I, Ninkovic M, Nedeljkovic N, Dragic M. Sustained Systemic Antioxidative Effects of Intermittent Theta Burst Stimulation beyond Neurodegeneration: Implications in Therapy in 6-Hydroxydopamine Model of Parkinson’s Disease. Antioxidants. 2024 Feb 8;13(2). doi:10.3390/antiox13020218

7. Glajch KE, Fleming SM, Surmeier DJ, Osten P. Sensorimotor assessment of the unilateral 6-hydroxydopamine mouse model of Parkinson’s disease. Behav Brain Res. 2012 May 1;230(2):309–16. doi:10.1016/j.bbr.2011.12.007 PubMed PMID: 22178078; PubMed Central PMCID: PMC3324279.

8. Hamadjida A, Frouni I, Kwan C, Huot P. Classic animal models of Parkinson’s disease: a historical perspective. Behav Pharmacol. 2019 Jun;30(4):291–310. doi:10.1097/FBP.0000000000000441 PubMed PMID: 30216234.

9. Yörük MA, Okkay U, Savaş AB, Bayram C, Sezen S, Ertuğrul MS, et al. Behavioral Tests Used in Experimental Animal Models. Anatol J Bio. 2022 Dec 31;3(2):2.

10. Sheta R, Bérard M, Musiol D, Martínez-Drudis L, Oueslati A. Behavioral analysis of motor and non-motor impairment in rodent models of Parkinson’s disease. Front Aging Neurosci. 2024 Dec 23;16:1464706. doi:10.3389/fnagi.2024.1464706 PubMed PMID: 39763579; PubMed Central PMCID: PMC11701160.

11. Prasad EM, Hung SY. Behavioral Tests in Neurotoxin-Induced Animal Models of Parkinson’s Disease. Antioxidants (Basel). 2020 Oct 16;9(10):1007. doi:10.3390/antiox9101007 PubMed PMID: 33081318; PubMed Central PMCID: PMC7602991.

12. Wang X, Han D, Zheng T, Ma J, Chen Z. Modulation of human induced neural stem cell-derived dopaminergic neurons by DREADD reveals therapeutic effects on a mouse model of Parkinson’s disease. Stem Cell Res Ther. 2024 Sep 11;15(1):297. doi:10.1186/s13287-024-03921-y PubMed PMID: 39256801; PubMed Central PMCID: PMC11389507.
