# Supplementary material for: Modulation of Oxidative Stress and Apoptosis by Antrodia cinnamomea–Loaded Citrate-Stabilized Silver Nanoparticles in Experimental Parkinsonism
Source: Mol Neurobiol. 2026 Apr 21;63(1):576. doi: 10.1007/s12035-026-05853-5 (PMC13099803; doi:10.1007/s12035-026-05853-5)

Western Raw band:

Control AC AgNPs AC+AgNPs L-DOPA+6-OHDA 6-OHDA AC+6-OHDA AgNPs+6-OHDA AC+AgNPs+6-OHDA

1-Agmatinase:

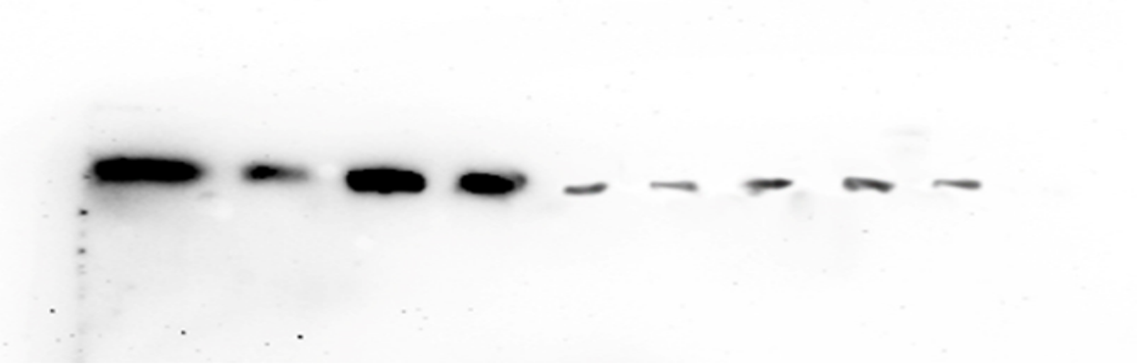

2-Bcl-2:

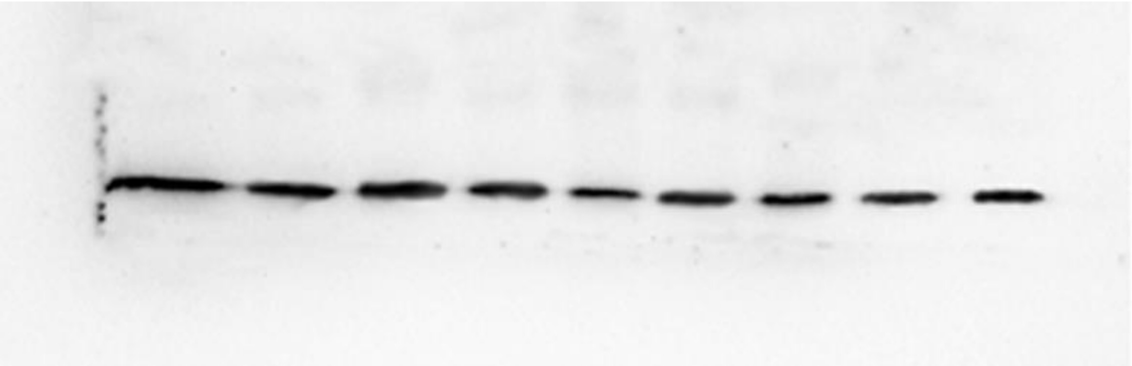

3-Caspase-3:

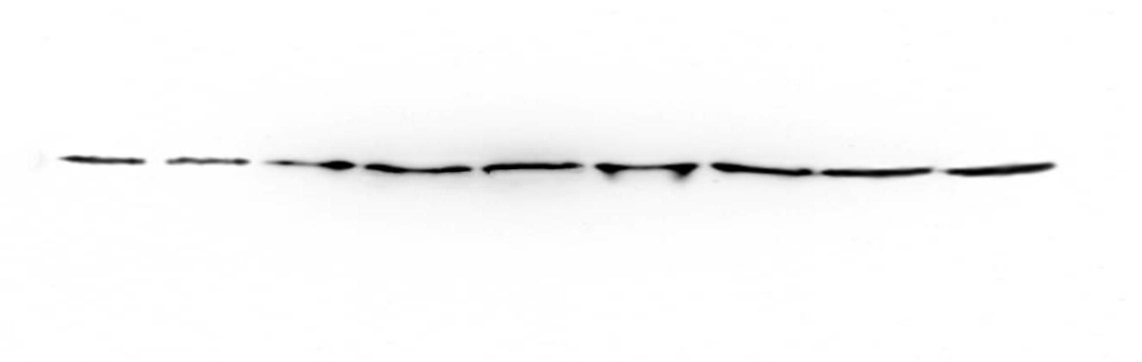

4-Tyrosine:

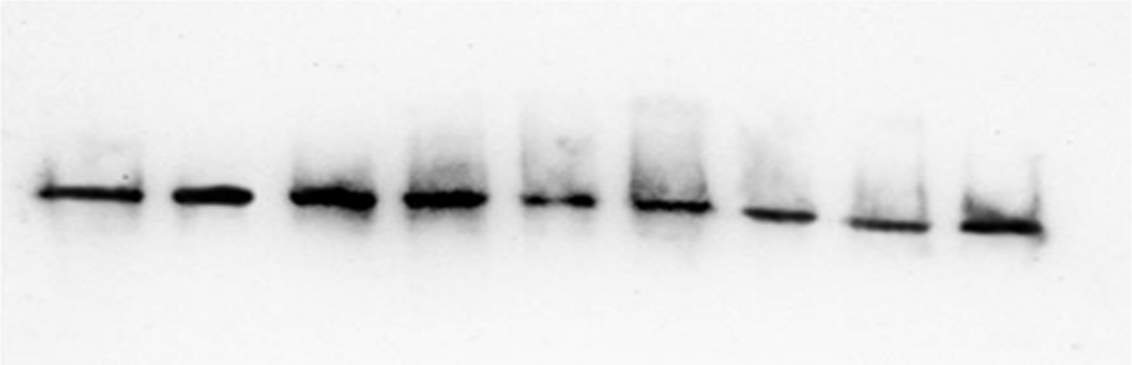

5-PI3K:

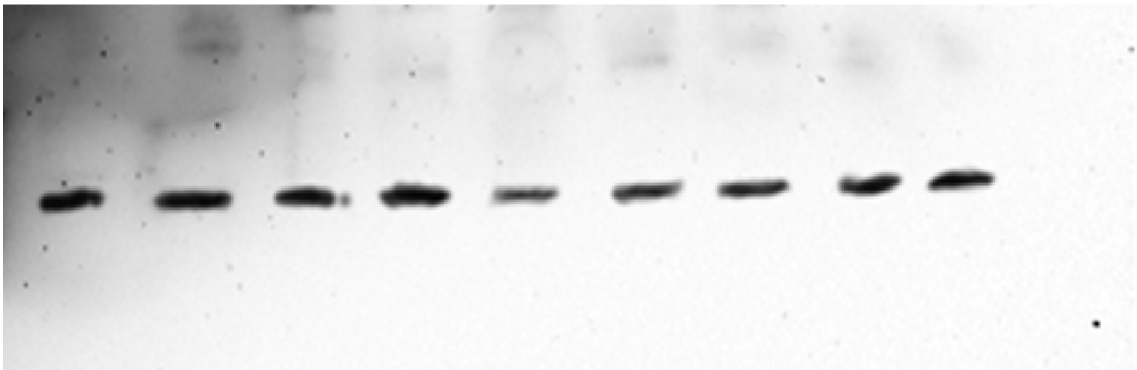

6-Beta actin:

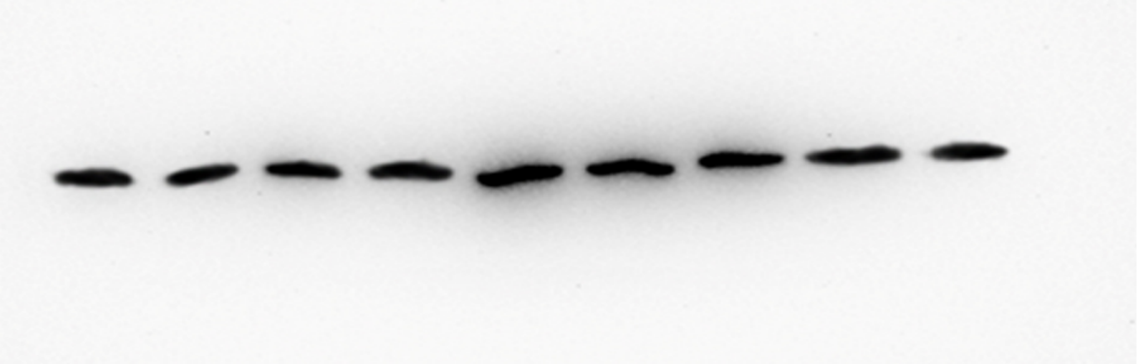

Supplement: Supplementary file 2 — (PDF 420 KB) [file 12035_2026_5853_MOESM2_ESM.pdf]
